# Weather deviations linked to undocumented migration and return between Mexico and the United States

**DOI:** 10.1073/pnas.2400524121

**Published:** 2024-11-04

**Authors:** Julia Li Zhu, Nancy Chau, Amanda D. Rodewald, Filiz Garip

**Affiliations:** ^a^Department of Economics, San Diego State University, San Diego, CA 92182; ^b^Charles H. Dyson School of Applied Economics and Management, Cornell University, Ithaca, NY 14853; ^c^Natural Resources and the Environment and Laboratory of Ornithology, Cornell University, Ithaca, NY 14853; ^d^Department of Sociology and School of Public and International Affairs, Princeton University, Princeton, NJ 08544

**Keywords:** return migration, climate change, undocumented migration, weather extremes, Mexico

## Abstract

As the world’s climate continues to change, human populations are exposed to increasingly severe and extreme weather conditions that can promote migration. Here, we examine how extreme weather influences the likelihood of undocumented migration and return between Mexico and the United States. We used data from 48,313 individuals observed between 1992 and 2018 in 84 Mexican agricultural communities. While controlling for regional and temporal confounding factors, we related individual decisions to migrate to the United States without documents and subsequently return to Mexico with lagged weather deviations from the historical norm during the corn-growing season (May to August). Undocumented migration was most likely from areas experiencing extreme drought, and migrants were less likely to return to their communities of origin when extreme weather persisted. These findings establish the role of weather shocks in undocumented Mexican migration to, and eventual settlement in, the United States. The findings also suggest that extreme weather conditions, which are likely to increase with climate change, promote clandestine mobility across borders and, thus, expose migrants to risks associated with crossing dangerous terrain and relying upon smugglers.

## Significance

Over two million undocumented migrants originating from Mexico or other countries crossed the Mexico–U.S. border in fiscal year 2022, many of them putting their lives at risk. Our work shows that weather shocks in Mexico, along with well-established economic and social conditions, are historically linked to this clandestine mobility. Specifically, Mexicans who depend on rain-fed agriculture are more likely to migrate to the United States without documents after extremely dry seasons. Weather also plays a role in migrants’ decisions to return to Mexico. Undocumented Mexicans are more likely to stay in the United States if their communities continue to experience extreme rainfall. These findings underscore the importance of recognizing weather-driven mobility, which can increase with anthropogenic climate change.

Weather patterns shape both human settlement and mobility (1). Weather-related disasters, like Hurricane Katrina in the Gulf Coast, can displace millions overnight (2). Gradual weather changes, such as persistent droughts in Ethiopia, can trigger migration out of regions dependent on rain-fed agriculture (3). These weather shifts are expected to intensify with anthropogenic climate change and contribute to large-scale human mobility (4).

Research shows that the extent to which individuals migrate can be affected by both sudden weather events (5), like tornadoes (6), hurricanes (2), and floods (7), and slow-onset weather processes, such as above average temperatures (8) or deviations in rainfall (9). Most weather-related migration occurs within national borders given high costs of international movement (10). That said, weather stressors can promote international migration in cases where origin–destination countries are connected via migrant networks, and when clandestine crossing is possible, as is the case between Mexico and the United States (11) (*SI Appendix*, *Weather and Migration*).

We focus on the migration between Mexico and the United States, the largest sustained international flow in the world. By 2017, over 10 million Mexican-born individuals were residing in the United States, about half of them without documents (12). We study this latter group. Undocumented migrants not only face risks as they move across unfamiliar and often hostile terrain but also from working stressful, dangerous, unhealthy, and low-paying jobs once in the United States (13, 14). After peaking at 6.9 million in 2005, the number of undocumented Mexicans in the United States dropped to 4.9 million by 2017. Yet the share of undocumented Mexicans who have been in the country for 10 y or more has increased from 41% in 2005 to 83% in 2017 (15). Our goal is to link weather conditions to the likelihood and duration of undocumented mobility from Mexico to the United States.

Mexico is a key setting for studying the potential impact of weather stressors on migration. The country is projected to experience a 1.1-to-3-degree Celsius increase in mean annual temperature by 2060, in addition to the 0.6 degree increase it has already experienced from 1960 to 2003 (16). Weather extremes are likely to create significant economic damage for rural populations, the majority of which are dependent on rain-fed agriculture given that only a quarter of cultivated land is irrigated (17). Weather extremes accounted for approximately 80% of economic losses due to natural disasters in Mexico between 1980 and 2005 (17), a share that is likely to increase with the expected climate change impacts (4).

Mexico is also an important case for investigating return migration. The country received the largest return flow in the world in 2010 to 2015 (18), with 1.3 million Mexicans returning from the United States in this period in addition to the 1.4 million who had returned in 2005 to 2010 (19). Research generally points to family reunification (19–21), restrictive immigration policies (19), and economic conditions [e.g., losing a job (19) or reaching a savings target (22)] as the main reasons for return from United States to Mexico, but the extent to which weather extremes contribute to return migration remains poorly understood.

We examine how weather fluctuations relate to both undocumented migration from, and return migration to, Mexico. This dual focus allows us to investigate undocumented migration not as a discrete choice but as a process that unfolds over time. We posit two hypotheses. First, we expect the likelihood of undocumented migration to be higher when agricultural communities experience weather shocks that deviate from historical patterns, all else equal. Second, we expect the likelihood of return migration to be lower if weather deviations persist in migrants’ origin communities.

Migration is an economic and social process. Prominent theories describe migration as a strategy to increase earnings (23) and diversify risks (24) that is facilitated by network effects whereby prior migrants offer information, help, or normative pressures that make migrating easier, leading to “cumulative causation” of migration (25). These network effects may be especially strong in communities with a history of migration.

Migration scholars often think of weather shocks as increasing risks to weather-dependent economic activities and thus triggering migration as a risk-diversification strategy (10). Our hypotheses are consistent with this view. Migration scholars also consider heterogeneity in weather-related migration. One source of variation is household wealth. On one hand, poor households might be more vulnerable to weather shocks, and hence, have a greater propensity to send migrants (26). On the other hand, poor households may lack sufficient resources to finance a costly move (27). In the Mexico–U.S. case, we expect the latter pattern to dominate. Any positive relationship between weather shocks and undocumented migration should be stronger for wealthier households. Likewise, we expect that weather shocks will more easily trigger migration in communities with a higher prevalence of migration.

We test these hypotheses using data from the Mexican Migration Project (MMP), a repeated cross-sectional survey from 170 communities in migrant-sending areas of Mexico, combined with gridded daily precipitation and temperature data from Daymet. The MMP data are not representative of the Mexican population but provide an accurate profile of U.S.-bound migrants that is consistent with benchmark national datasets (28). Because our dataset includes timing and duration of migration trips, we can precisely track individual-level migration and return behaviors.^*^ Restricting our analysis to 48,313 individuals residing in 84 agricultural communities, we connect precipitation and temperature deviations from the historical community average to subsequent decisions to cross the U.S. border without documents, and to eventually return to Mexico, over a 27-y (1992 to 2018) period (*Materials and Methods*).

**Fig. 1.**
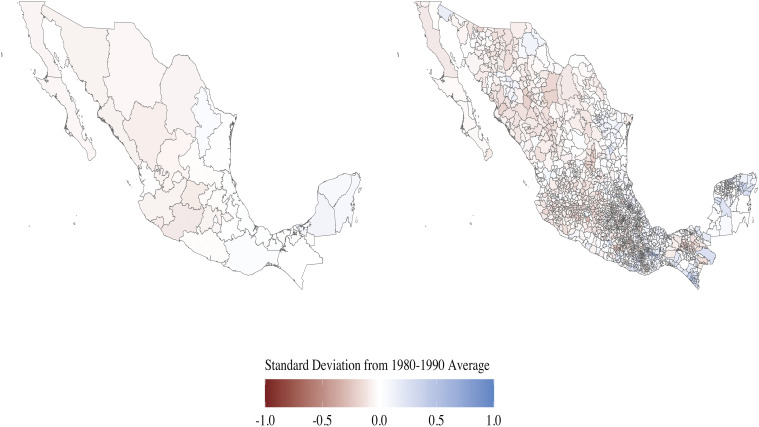
Rainfall deviation in state (*Left*) and municipality (*Right*) equals average daily rainfall in 2005 minus average rainfall in baseline period (1980 to 1990), divided by SD in rainfall in that period. Red hues indicate that a state was drier in 2005 relative to 1980 to 1990; blue hues show that it was wetter.

We measure weather at the community level. Prior work measures weather at the municipality (30) or state (31) level. Fig. 1 presents precipitation deviation in Mexican states (*Left*) and municipalities (*Right*) to show the implications of the spatial unit of analysis. The deviation equals the average precipitation in 2005 minus the average precipitation in baseline period (1980 to 1990), divided by the SD of precipitation in that period. Red hues indicate a state was drier in 2005 relative to 1980 to 1990; blue hues show that it was wetter. State-level precipitation measure (*Left*) hides variation across municipalities in each state. Municipality-level measure (*Right*), similarly, conceals variation across communities in our data (which are too small to show on a map).

Existing research on the weather–migration link in Mexico presents mixed results (*SI Appendix*, *Weather and Migration in Mexico*). The inconsistencies across studies reflect contextual variation as well as different strategies for measuring weather and for linking that change to migration (*SI Appendix*, *Linking Weather to Migration*). Our approach offers improvements to existing work. First, we start with fine-grained weather data measured daily and at 1 km^2^ resolution. We aggregate these data at the community level to capture spatial variations that are averaged out in coarser measures. Second, we measure migration behavior at the individual level and annually rather than in aggregate counts (32) or across wider time windows (30). Third, we consider a particular (agricultural) mechanism underlying the weather–migration link rather characterizing a general relationship. We only investigate communities where majority of the population depends on agriculture and customize weather measures to capture the weather sensitivity of the most common crop (corn) during its growing season. Fourth, we use a stringent empirical specification that partials out state and year fixed effects, thus focusing attention on the variation between communities in a state in a year, and control for potential confounders at the individual and household level.

## Results

### Weather and Undocumented Migration.

Our first outcome of interest is whether an individual takes their first undocumented trip to the United States in a year. We focus on the first trip to avoid concerns about path dependence. Prior trips might bring changes to the migrant (e.g., more ties to destination and more knowledge about crossing pathways and costs) or household (e.g., remittances’ expectations) that make future migration more likely and less dependent on other factors, including weather shocks. We consider this possibility in robustness checks. To model the migration outcome within the multilevel data structure, we estimate a linear probability model with state and year fixed effects and cluster-robust SEs to account for within-community correlations in migration choices. Our model links weather fluctuations to undocumented migration as follows.

[1] $$y_{\mathrm{ijcst}}=\beta^{\prime }w_{cst-1}+\delta^{\prime }x_{\mathrm{ijcst}}+\nu^{\prime }h_{jcst-1}+\gamma^{\prime }z_{cst-1}+\alpha_{s}+\omega_{t}+\varepsilon_{\mathrm{ijcst}}$$

$y_{\mathrm{ijcst}}$ denotes the binary outcome of whether an individual *i* from household *j* in community *c* in state *s* took an undocumented first trip to the United States in year *t*. The vector $w_{cst-1}$ represents weather (precipitation and temperature) indicators for community *c* in year *t*-1, measured as normalized deviations from the community annual averages in the decade prior to analysis period (1980 to 1990). The vector *β* represents the coefficients for weather deviations, prime (^′^) denotes transpose. The vector $x_{\mathrm{ijcst}}$ includes individual characteristics that are likely to be related to migration choices. In Mexico, the likelihood of undocumented migration is higher among younger adults who can take on the migrant journey and send remittances while delegating responsibilities at home to their older siblings or parents (33). Due to traditional gender norms and structure of labor demand in the United States, men migrate more than women (33). Given their lack of access to urban jobs, individuals with low education are more likely to cross the border than those with high education (34). The coefficient vector δ captures these patterns in our data

The vector $h_{jcst-1}$ contains time-varying household attributes measured at *t*-1. Given the financial costs of border crossing, undocumented migrants in Mexico typically come from middle-income households that can afford the smuggling fees (34). Undocumented migrants also often originate from households with prior migration experience. The coefficient ν represents these relationships between household attributes (land, business and property ownership, prior migration experience) and undocumented migration.

The vector $z_{cst-1}$ denotes time-varying community characteristics at *t*-1. Weather shocks often influence migration decisions through their effect on agricultural production (32). Studies observe weather-driven migration responses in communities that are rural and those that are heavily involved in agriculture (35). In line with the cumulative causation idea, research repeatedly finds high migration rates out of communities where migration is already prevalent (34). Finally, research considers community distance to the Mexico–U.S. border as a potential determinant of migration. The coefficient vector γ captures the effect of these community-level factors (share of men working in agriculture, share of individuals who have ever migrated to the United States, distance of community to border) on undocumented migration. The terms $\alpha_{s}$ and $\omega_{t}$represent the fixed effects for state *s* and time *t*, respectively; $\varepsilon_{\mathrm{ijcst}}$ is the error term.

The inclusion of state and year fixed effects makes this specification particularly powerful. The fixed effects absorb, for example, any macrolevel shocks (e.g., in exchange rates) and policy shifts. The fixed effects also eliminate any unmeasured variation across states, like agricultural output or policies. (*SI Appendix*, *Implications of the Fixed Effects Specification*).

Table 1 presents our results. The models are estimated on data (425,062 person-years) from 84 communities where 50% or more of men work in agriculture. Corn is the most common crop in these communities, accounting for 43% of harvested land on average. Corn yields are sensitive to rainfall (36) and temperature (37) extremes during the growing season (May to August) (*Materials and Methods*). By focusing on agricultural communities, we presume that weather–migration link works mostly through declining crop yields, although we do not test this mechanism directly.

**Table 1.** Coefficient estimates for weather indicators from linear probability models of first undocumented migration in the MMP data

Weather last year	(1) Weather in season (May to Aug)	(2) Weather outside season (<May or >Aug)	(3) Temperature measured in GDD	(4) Outcome includes working tourists	(5) Outcome includes documented migrants
Very dry	0.0030*	−0.0013	0.0030*	0.0029*	0.0035*
	(0.0013)	(0.0017)	(0.0013)	(0.0012)	(0.0015)
Dry	0.0000	−0.0012	−0.0000	0.0000	0.0000
	(0.0006)	(0.0008)	(0.0006)	(0.0007)	(0.0006)
Wet	0.0001	0.0009	0.0001	0.0001	−0.0004
	(0.0008)	(0.0006)	(0.0008)	(0.0008)	(0.0008)
Very wet	0.0018	0.0012	0.0018	0.0015	0.0009
	(0.0010)	(0.0009)	(0.0010)	(0.0010)	(0.0010)
Very cool	−0.0007	0.0019	−0.0008	−0.0009	−0.0010
	(0.0012)	(0.0011)	(0.0012)	(0.0012)	(0.0012)
Cool	0.0005	0.0013	0.0005	0.0005	0.0001
	(0.0008)	(0.0011)	(0.0008)	(0.0008)	(0.0008)
Hot	0.0001	0.0008	0.0000	0.0000	−0.0002
	(0.0006)	(0.0007)	(0.0006)	(0.0006)	(0.0006)
Very hot	0.0004	0.0010	0.0005	0.0005	0.0005
	(0.0013)	(0.0009)	(0.0013)	(0.0013)	(0.0014)

N (person-years)	425,062	425,062	425,062	426,150	428,497
$R^{2}$	0.0124	0.0124	0.0124	0.0124	0.0129

**P < 0.01, *P < 0.05. GDD = Growing Degree Days. Robust SEs in parentheses. Normal weather is the reference. A community is very wet (very dry) if rainfall is over two SDs higher (lower) than its baseline (1980 to 1990) mean, wet (dry) if rainfall is one-to-two SDs higher (lower), and normal otherwise. Models control for individual (age, sex, education), household (land, business, and property ownership, prior US migrants), and community characteristics (share in agriculture, share of ever migrants, distance to US border) as well as state and year dummies.

Of the 48,313 unique individuals in our data, 3,723 (8%) have crossed the border without documents for the first time in the study period, 181 (0.4%) have worked with a tourist visa (thus violating the conditions of the visa), and 501 (1%) have entered and remained with documents. Main analysis focuses on the first (undocumented entry) group. Our key indicators capture weather deviations from the historical community average (1980 to 1990) during corn-growing season (May to August) in previous year. A community is considered very wet (very dry) if rainfall is over two SDs above (below) its historical average, wet (dry) if rainfall is one-to-two SDs higher (lower), and normal otherwise. Temperature deviations (based on daily average temperatures from May to August) are computed similarly. Because we control for state and year dummies, the estimates capture weather deviation effect within states after controlling for any temporal shocks.

Column 1) shows coefficient estimates for rainfall and temperature extremes during corn season (May to August); the outcome is first undocumented entry to the United States. (*SI Appendix*, Table S3 shows the remaining coefficient estimates.) The estimates offer support for our first hypothesis, but show variation across weather conditions. Very dry weather has a statistically significant association (P<0.05) with a higher likelihood of undocumented crossings in subsequent year; other rainfall and temperature extremes do not. Column 2) repeats the same analysis with weather conditions measured outside the corn season (before May and after August). None of the weather extremes obtain statistically significant coefficients. Rainfall shortages that occur within the season matter; those outside the season do not. This pattern reinforces the presumed agricultural linkage between weather and undocumented migration.

Column 3) introduces an alternative measure of temperature shocks based on growing degree days (GDD). Corn yields vary with temperature in a nonlinear fashion, peaking at moderate values, and declining after 30 °C (37). To capture this pattern, we convert daily temperatures (T) to GDD according to Eq. **2** and sum over the growing season. For each community-year, we compute the deviations in seasonal (May-to-August) GDD from the baseline period (1980 to 1990). The results in Column 3) remain identical to 1); GDD deviations show no statistically significant association to undocumented crossings.

[2] $$\begin{array}{c} \mathrm{GDD}\left(T\right)=\left\{\begin{array}{cc} 0, & \;\text{if}\;T\leq {\text{8}}^{{}^{\circ}}\;\text{C}\\ T-8, & \;{\text{8}}^{{}^{\circ}}\;\text{C}<T\leq {\text{30}}^{{}^{\circ}}\;\text{C}\\ 22, & \;\text{if}\;T>{\text{30}}^{{}^{\circ}}\;\text{C} \end{array}\right. \end{array}$$

The remaining columns show results for alternative outcomes. Column 4) combines migrants who cross without documents with those who work in the United States despite entering with a tourist visa. Column 5) adds documented migrants to the mix. In both cases, the coefficients for in-season weather extremes remain similar to Column 1). Within each state, communities experiencing extremely dry weather show higher migration rates in the following year relative to communities receiving normal rainfall.

The link between weather shocks and migration can vary by household wealth. Given costs associated with crossing the Mexico–U.S. border, we expect a stronger relationship between weather extremes and undocumented migration among wealthier households. To test this idea, we separately analyzed households with no assets (about a fourth of the sample) and those owning some land, or a business, or a property. The results for undocumented migration are presented in the first two models in Table 2. As expected, we observe a positive link (P< 0.05) between very dry weather and undocumented migration among households with assets, but not among those with no assets.

**Table 2.** Coefficient estimates for weather indicators from linear probability models of first undocumented migration estimated on samples varying by household wealth and community migration experience in the MMP data

Weather last year	Household owns land, business or property		Share ever migrated in community	
	No	Yes	Low	High
Very dry	0.0004	0.0039**	−0.0002	0.0085*
	(0.0039)	(0.0015)	(0.0013)	(0.0035)
Dry	0.0016	−0.0004	−0.0003	−0.0006
	(0.0012)	(0.0006)	(0.0006)	(0.0012)
Wet	0.0006	−0.0001	0.0010	−0.0016
	(0.0013)	(0.0008)	(0.0008)	(0.0012)
Very wet	0.0048*	0.0009	0.0008	−0.0008
	(0.0019)	(0.0012)	(0.0009)	(0.0014)
Very cool	−0.0017	−0.0004	0.0003	−0.0041
	(0.0027)	(0.0013)	(0.0014)	(0.0024)
Cool	−0.0009	0.0009	0.0013	−0.0002
	(0.0014)	(0.0008)	(0.0006)	(0.0016)
Hot	0.0018	−0.0004	0.0001	−0.0006
	(0.0014)	(0.0007)	(0.0007)	(0.0011)
Very hot	0.0032	−0.0003	0.0006	−0.0031
	(0.0022)	(0.0014)	(0.0014)	(0.0021)

N (person-years)	92,700	332,362	244,524	180,538
$R^{2}$	0.0124	0.0128	0.0102	0.0151

**P < 0.01, *P < 0.05. Robust SEs in parentheses. Normal weather is the reference. A community is very wet (very dry) if rainfall is over two SDs higher (lower) than its baseline (1980 to 1990) mean, wet (dry) if rainfall is one-to-two SDs higher (lower), and normal otherwise. Models control for individual (age, sex, education), household (land, business, and property ownership, prior US migrants), and community characteristics (share in agriculture, share of ever migrants, distance to US border) as well as state and year dummies.

Resource constraints to migration might be less binding in communities with an established history of migration, where prior migrants provide information and help that reduce the costs of crossing the border or finding a job in the United States (25). Therefore, we expect a stronger link between weather extremes and undocumented migration in communities with higher migration prevalence. In the sample communities, migration prevalence (percentage of prior U.S. migrants) ranges from 0.2% to 57%. Using the median value (12%), we divided our sample into communities with low versus high migration prevalence. The results are presented in the last two models in Table 2. Consistent with expectations, we find that the positive association between very dry weather and undocumented migration holds only in communities with high migration prevalence (with >12% ever migrants in t-1).

### Weather and Return among Undocumented Migrants.

Our second outcome is whether an undocumented migrant in the United States returns to Mexico. The model structure is like Eq. **1**; $y_{\mathrm{ijcst}}$ now denotes whether an undocumented migrant *i* from household *j* in community *c* in state *s* returned home in year *t*. The model includes the same indicators as before; but introduces dummies for California, Illinois, and Texas (top three states attracting about 60% of migrants) to account for unobserved time-invariant destination-specific variation. Year dummies absorb any variation in return migration incentives due to U.S. border enforcement efforts over time.

Research shows that migration duration can be dictated by costs incurred to finance the move. Undocumented migrants that rely on a *coyote* (smuggler) are likely to stay longer in the United States (38). Information on coyote use is missing for 75% of our sample. Among the quarter with intact information, 93% report crossing with a coyote on their first trip. We do not include this indicator in our models to retain sample size; we presume that household wealth indicators (land, business, and property ownership) will capture the potential debt burden (or lack thereof) from hiring coyotes. Because migrant wages in the United States were also missing for 69% of our sample, we instead include an indicator for whether migrant works in agriculture (as opposed to higher-paying manufacturing or service jobs).

Table 3 presents coefficient estimates for rainfall and temperature extremes in undocumented migrants’ origin community during migrant’s first year in the United States. (The coefficients for the remaining indicators are listed in *SI Appendix*, Table S3.) The outcome is whether a migrant returns to Mexico within their second year in destination. The data come from N = 2,765 migrants who have crossed without documents, all observed after their first year in the United States.

**Table 3.** Coefficient estimates for weather indicators from linear probability models of return migration within the second year in the United States in the MMP data

Weather last year	(1) Weather in season (May–Aug)	(2) Weather outside season (<May or >Aug)	(3) Temperature measured in GDD	(4) Sample includes working tourists	(5) Sample includes documented migrants
Very dry	−0.1197**	0.0863	−0.1199**	−0.1182**	−0.1164**
	(0.0307)	(0.1629)	(0.0307)	(0.0306)	(0.0296)
Dry	0.0115	0.0196	0.0113	0.0061	0.0095
	(0.0380)	(0.0308)	(0.0379)	(0.0383)	(0.0374)
Wet	−0.0896**	0.0006	−0.0900**	−0.0903**	−0.0839**
	(0.0289)	(0.0396)	(0.0289)	(0.0296)	(0.0299)
Very wet	−0.0805*	−0.0700*	−0.0794*	−0.0771*	−0.0730*
	(0.0335)	(0.0300)	(0.0336)	(0.0342)	(0.0347)
Very cool	0.0366	−0.0318	0.0460	0.0393	0.0350
	(0.0722)	(0.0350)	(0.0721)	(0.0760)	(0.0727)
Cool	−0.0098	−0.0324	−0.0137	−0.0118	−0.0121
	(0.0217)	(0.0319)	(0.0214)	(0.0225)	(0.0226)
Hot	0.0114	−0.0237	0.0086	0.0135	0.0130
	(0.0302)	(0.0248)	(0.0298)	(0.0302)	(0.0302)
Very hot	0.0949	0.0114	0.0936	0.0731	0.0698
	(0.0646)	(0.0349)	(0.0644)	(0.0612)	(0.0591)

N (persons)	2,765	2,765	2,765	2,862	3,034
$R^{2}$	0.1461	0.1373	0.1464	0.1403	0.1387

**P < 0.01, *P < 0.05. GDD = Growing Degree Days. Robust SEs in parentheses. Normal weather is the reference. A community is very wet (very dry) if rainfall is over two SDs higher (lower) than its baseline (1980 to 1990) mean, wet (dry) if rainfall is one-to-two SDs higher (lower), and normal otherwise. Models control for individual (age, sex, education, U.S. occupation), household (land, business, and property ownership, prior US migrants), and community characteristics (share in agriculture, share of ever migrants, distance to US border) as well as Mexican state, U.S. state, and year dummies.

Results in column 1) are in line with our second hypothesis. Extreme seasonal rainfall (very dry, wet, or very wet conditions) in origin communities has a statistically significant association (P< 0.05) with a lower likelihood of return among undocumented migrants from those communities; extreme temperature does not. Weather shocks outside the corn-growing season are not statistically linked to rates of return (with the exception of very wet conditions) as shown in column 2). Seasonal rainfall coefficients remain robust to measuring temperature with GDD in column 3) and to using alternative samples that include migrants working on tourist visas in column 4) or documented migrants in column 5).

About 16% (N= 585) of undocumented migrants return within their first year in the United States. We excluded this group from the analysis above given that the same weather conditions (that is, in year t-1) are likely associated with both undocumented migration and return (in year t). The results remain similar, however, when we replicate the analysis for this group. (*SI Appendix*, Table S4.) About 16% of undocumented migrants return after staying a year in the United States. 16% return after staying two years, 11% after three years, and 8% after four years. Weather conditions might matter less in return decisions as migrants establish roots in the United States.

To capture this possibility, we estimate a series of models. The first model in Table 4 includes seasonal rainfall and temperature deviations in undocumented migrants’ origin community during migrant’s second year in the United States. The outcome is whether a migrant returns to Mexico within their third year in destination. The second model includes weather deviations in origin during migrants’ third year; the outcome is return during their fourth year. The last column similarly shifts the time horizon to migrants’ fourth year in the United States and considers return in their fifth year. The sample size becomes too small to consider further out years.

**Table 4.** Coefficient estimates for weather indicators from linear probability models of return migration within third, fourth, and fifth years in the United States in the MMP data

Weather	Return within		
last year	Third year	fourth year	Fifth year
Very dry	−0.1250	−0.0942	−0.1693**
	(0.0631)	(0.0502)	(0.0544)
Dry	0.0155	0.0538	0.0678
	(0.0357)	(0.0532)	(0.0475)
Wet	−0.1422**	−0.1080**	−0.1121**
	(0.0319)	(0.0303)	(0.0397)
Very wet	−0.1181**	−0.1066*	−0.1111
	(0.0307)	(0.0488)	(0.0667)
Very cool	0.1855	0.2300	0.1199
	(0.1067)	(0.1449)	(0.1401)
Cool	−0.0259	−0.0273	−0.0632
	(0.0280)	(0.0543)	(0.0507)
Hot	0.0229	0.0607	0.0256
	(0.0389)	(0.0504)	(0.0586)
Very hot	0.0345	0.0147	−0.0454
	(0.0681)	(0.0616)	(0.0608)

N (persons)	2,180	1,740	1,424
$R^{2}$	0.2270	0.2886	0.3327

**P < 0.01, *P < 0.05. GDD = Growing Degree Days. Robust SEs in parentheses. Normal weather is the reference. A community is very wet (very dry)if rainfall is over two SDs higher (lower) than its baseline (1980 to 1990) mean, wet (dry) if rainfall is one-to-two SDs higher (lower), and normal otherwise. Models control for individual(age, sex, education, U.S. occupation), household (land, business, and property ownership, prior US migrants)and community characteristics (share in agriculture, share of ever migrants, distance to US border) as well as Mexican state, U.S. state, and year dummies.

The results paint a consistent picture, linking extreme weather conditions in origin communities to a lower likelihood of return. Individuals from communities experiencing very dry weather are less likely to return in the following year compared to individuals from communities in the same state with normal weather. This relationship is below the 95%-threshold for statistical significance for return within fifth year, and very close to it for return within third (P= 0.051) and fourth (P= 0.064) years. Individuals from communities with wet or very wet weather (as opposed to normal weather) are also less likely to return in the following year. This pattern holds regardless of the time migrants have remained in the United States. While undocumented migration seems only related to rainfall deficits (Table 1), return migration is negatively linked to more varied extreme conditions, including excess rainfall. These results are similar to Entwisle et al.’s (39) findings in Thailand, where weather shocks had small positive effects on outmigration from rural villages to urban areas, but significant negative effects on return decisions to those villages.

As in the analysis of undocumented migration, we consider variations in the weather-return link by household wealth and community migration prevalence in Table 5. The negative association between rainfall extremes and return migration persists for very dry and wet conditions among households with assets and for very wet conditions among those lacking assets. In the Mexican setting, then, households with assets tend to delay return under both rainfall deficits and excess, while those with no assets do so only rainfall is excessive.

**Table 5.** Coefficient estimates for weather indicators from linear probability models of return migration within second year in the U.S estimated on samples varying by household wealth and community migration experience in the MMP data

Weather last year	Household owns land, business or property		Share ever migrated in community	
	No	Yes	Low	High
Very dry	−0.0991	−0.1257**	−0.1435**	−0.1856*
	(0.0529)	(0.0319)	(0.0509)	(0.0789)
Dry	−0.0545	0.0388	0.0504	−0.0076
	(0.0385)	(0.0454)	(0.0417)	(0.0562)
Wet	−0.0214	−0.0996*	−0.0358	−0.1256**
	(0.0394)	(0.0389)	(0.0263)	(0.0455)
Very wet	−0.1205*	−0.0608	−0.0371	−0.1282*
	(0.0530)	(0.0430)	(0.0377)	(0.0502)
Very cool	−0.0637*	0.0576	−0.0343	0.1077
	(0.0271)	(0.0859)	(0.0434)	(0.1330)
Cool	0.0276	−0.0198	0.0244	−0.0143
	(0.0382)	(0.0268)	(0.0287)	(0.0328)
Hot	0.0251	0.0076	−0.0139	0.0273
	(0.0297)	(0.0430)	(0.0445)	(0.0388)
Very hot	−0.0607	0.1437	−0.0637	0.1994*
	(0.0427)	(0.0814)	(0.0516)	(0.0946)

N (persons)	765	1,997	1,135	1,625
$R^{2}$	0.1362	0.1859	0.1644	0.1947

**P < 0.01, *P < 0.05. Robust SEs in parentheses. Normal weather is the reference. A community is very wet (very dry) if rainfall is over two SDs higher (lower) than its baseline (1980 to 1990) mean, wet (dry) if rainfall is one-to-two SDs higher (lower), and normal otherwise. Models control for individual (age, sex, education, U.S. occupation), household (land, business, and property ownership, prior US migrants), and community characteristics (share in agriculture, share of ever migrants, distance to US border) as well as Mexican state, U.S. state, and year dummies.

The last two columns show patterns in communities with low and high migration prevalence, respectively. The negative association between extreme rainfall and return migration persists across all conditions (for very dry, wet, and very wet conditions) in high prevalence communities, but only partially (for very dry conditions) in low prevalence communities. These results suggest that existing migrant networks are linked to both higher likelihood of weather-related undocumented mobility to, and longer duration of stay in, the United States.

### Robustness Checks and Extensions.

We restricted our analysis to the communities where at least 50% of men work in agriculture. Our results are similar when we relax this restriction. *SI Appendix*, Tables S5 and S6 show coefficients for seasonal weather extremes in models of undocumented migration and return, respectively. The models are estimated on alternative samples. The first model includes all communities (where 46% of men work in agriculture, at median). The second (third) model drops communities where less than 30% (40%) of men work in agriculture. The fourth model replicates our main analyses estimated on communities with half or more men in agriculture. The results are consistent across the four models. Very dry weather is positively associated with undocumented migration from a community (P< 0.05) (*SI Appendix*, Table S5); very dry, wet, and very wet weather are negatively linked to return migration to that community (P< 0.05) (*SI Appendix*, Table S6). The coefficient estimates appear larger in more restricted samples, but the differences are not statistically significant (P> 0.05). These patterns are still in line with the presumed agricultural mechanism given the high rates of agricultural employment (46% in the median community-year) in the unconstrained sample (first model). Indeed, when we run our models in the least agricultural communities (where less than 30% of men work in agriculture), we do not observe a statistically significant relationship between weather and undocumented migration (results available from authors).

We considered if cumulative weather conditions prior to migration might have a bearing on the trip duration. For example, repeated events in a community might lead migrants to plan for a longer stay. To test this idea, we included indicators for the share of years with extreme (nonnormal) rainfall and temperature in a community up until a migrant’s departure. These indicators did not obtain statistically significant coefficients, and other results remained similar (available from authors).

We considered if availability of irrigation in community reduces weather effects on migration and return. The MMP data record the area of irrigated and rain-fed land in the survey year in each municipality (within which each community is nested). This information is missing in 20 out of 84 communities. We measure the share of irrigated land in the remaining 64 communities, and assume it remains fixed over time. We then estimate our models of migration (*SI Appendix*, Tables S7) and return (*SI Appendix*, Tables S8) on alternative samples. The median community in our data has 15% of its land irrigated. The first model includes communities where at least 5% of land is irrigated. The remaining models include communities where at least 10%, 15%, 20%, and 25% of land is irrigated, respectively.

The results for migration (*SI Appendix*, Tables S7) show an irrigation-dependent pattern. Very dry weather is positively related (P< 0.05) to undocumented migration in the first two models, where irrigation threshold is low. The coefficient is just below (P= 0.07) our significance threshold in the third model, where sample communities are above the median (0.15) irrigation level. This relationship is far from statistical significance in the last two models, where sample communities have at least a fifth and fourth of their land irrigated, respectively. This pattern suggests that our main result in Table 1 is driven mainly by low-irrigation communities.

The results for return (*SI Appendix*, Table S8) paint a similar picture. Very dry weather is no longer associated with return migration (P< 0.05) in communities with 15% or more irrigated land. Wet weather is not related to return in places with over 20% irrigated land. Very wet weather remains negatively associated (P< 0.05) with return across all irrigation thresholds. (The significance level for 20% or more irrigation is P= 0.051.) Overall, the weather-return relationship seems to get weaker with increasing irrigation levels in the community.

Our analysis focused on first migration and return trip. Research on Mexico–U.S. migration shows that factors associated with taking a first trip might matter less for subsequent trips (34). To consider this idea, we estimate models of undocumented migration and return that consider both first and last migration trips. The MMP data record these trips consistently for all respondents. 93% of individuals make 2 trips or less. Like prior work, we control for prior migration experience in our models. The results in *SI Appendix*, Tables S9 show that weather has a weaker association with undocumented migration when repeat trips are included (model 1). The coefficient for very dry conditions (0.0027, P< 0.1) falls below the 95%-threshold for statistical significance. The results for return migration remain similar to those in Table 3 when we include migrants on repeat trips (model 2). Notably, migrants on a repeat trip are significantly less likely to return than their counterparts on a first trip (−0.33, P< 0.05).

Our models include year fixed effects that absorb trends in migration. National statistics suggest that net migration from Mexico to the United States (i.e., entries minus returns) dropped to zero in 2005 to 2014 (40) after remaining positive through the 1990s. Net migration turned positive again after 2014 due to lower rates of return (41). Previous studies have attributed declining entries from Mexico to lower fertility rates (42) and better employment options in Mexico (43), and fewer economic prospects in the United States after the Great Recession (43), whereas declining returns to Mexico are linked to stricter border enforcement that makes circular mobility difficult (44).

Our models capture the general trend in migration and return but cannot decompose it into its components. *SI Appendix*, Figs. S3 and S4 show the coefficient estimates for the year dummies in the models for undocumented migration (model 1, Table 1) and return (model 1, Table 3), respectively. Undocumented migration is significantly more likely (P< 0.05) in years 1995, and 1998 to 2000, and less likely in 2008, 2009, 2011, 2012, and 2018, relative to 1992. Return is significantly (P<0.05) more likely in 1995 to 1997, 2003, and 2008, relative to 1992.

The weather impacts on migration and return are comparable in size to the year effects. According to our model, the dip in undocumented migration is largest in year 2012 (−0.0046, P< 0.05). The extreme-weather bump (0.0031 in very dry communities, P< 0.05) amounts to 67% (0.0031/0.0046) of the overall negative trend at its peak for individuals in exposed community-years (about 2% of the community-years in our data as shown in *SI Appendix*, Table S2). Our analysis offers a conservative estimate of the weather effect on migration. This estimate reflects the direct effect of weather once community-, state-, and year-specific factors are already accounted for.

## Discussion

Undocumented Mexican migrants have historically originated from rural areas, providing the agricultural labor that remained in short supply in the United States after the bilateral labor agreement, known as the Bracero Program, ended in 1964 (45). In recent decades, migrant origins have shifted to urban areas in Mexico and migrant occupations have diversified to manufacturing and service sectors in the United States (46). Our results link extreme weather conditions to continued undocumented migration out of rural Mexico.

Prior studies conducted at a wide range of temporal and regional scales report mixed findings on weather-related mobility from Mexico (*SI Appendix*, *Weather and Migration in Mexico*). According to a recent meta-analysis, studies that capture migration over short time spans, those that measure weather at fine geographic scales, and those that consider mechanisms of weather-related impacts produce stronger and more consistent evidence for weather-related mobility (47). We follow these practices in this study.

We combine gridded daily weather data with survey responses from 48,313 individuals between 1992 and 2018. We restrict our analysis to 84 rural Mexican corn-growing communities where weather shocks likely hurt weather-dependent agriculture. Our analysis links precipitation and temperature deviations from historical community averages during corn growing season to subsequent undocumented U.S. migration from, and eventual return to, Mexico. Our focus on return distinguishes our study from prior work (11, 30). We consider return as a choice that comes up each year a migrant spends in destination and estimate separate models across a migrant’s tenure in the United States. Thus, we treat undocumented migration not as a discrete event, but as a process where an individual needs to decide to become a migrant (or to remain one) in each year.

Our results show that weather shocks in origin communities are connected to both migration and return behaviors. Rainfall deficits during corn-growing season in a community (relative to the historical norm in 1980 to 1990) are related to increased likelihood of undocumented migration from that community in the subsequent year. This association is concentrated among households with some assets, who are in a better position to finance a costly move, and in communities with high migration prevalence, where ties to prior migrants facilitate the cross-border journey.

The weather–migration association is also stronger in communities with high levels of agricultural involvement and in municipalities with little irrigation. These patterns suggest that extreme weather likely impacts migration through its effect on agricultural yields. Investments in irrigation (which currently covers only a quarter of harvested area) (17) and insurance for weather-related loss might help us counter potential weather impacts on smallholder agriculture in Mexico and resulting undocumented migration to the United States.

The weather impacts on migration are small relative to those impacts on return decisions. In our case, relatively very dry, wet, or very wet seasons in a community seem to detract undocumented migrants from returning to that community the following year. We observe these patterns not only for recently arrived migrants but also for those who have been in the United States for two to four years. These results suggest that undocumented migrants continue to watch their communities and delay their return to Mexico if weather conditions fall outside their historical norm. Given that we consider precipitation and temperature in the preceding year alone, our results likely provide a lower bound to potentially longer-run weather effects on migration and return decisions.

To be sure, weather extremes are just one among many economic and social drivers of international migration. But these extremes are likely to increase with anthropogenic climate change and compound other drivers of human mobility (4, 48). The connection we identify between weather extremes and undocumented Mexican migration to (and prolonged stay in) the United States reminds us of what is at stake. Without alternatives in origin, or access to legal entry, Mexican migrants will continue to resort to dangerous and costly clandestine channels, and put their lives at risk to cross the border.

## Materials and Methods

### Migration Data.

The main data come from the MMP, a household survey from 170 communities in migrant-sending regions of Mexico (49). The MMP boasts low survey refusal rates (<5%), captures undocumented migrants (about 90% of migrants in the data) that are undercounted in the U.S. census, and records migration choices annually rather than in five-year windows like the Mexican census. The MMP team canvassed each community to randomly select 200 households and collected retrospective information on education, work, and migration experiences of all individuals including absent migrants. The team supplemented this information with records of household assets and community characteristics over time. The team also located migrants from the sample communities in the United States through their households in Mexico or referrals from other migrants. The referrals form a nonrandom sample and account for less than 2% of the data.

We downloaded the Mexican Migration Project data (MMP 170) from Princeton University’s Office of Population Research data archive (https://opr.princeton.edu/archive/mmp/). The MMP data are public and contain no individual identifiers; their use is exempt from IRB review. The data have a repeated cross-sectional design, where each community is surveyed once some time between 1982 and 2018. We combined different components of the data with individual (PERS), household (HOUSE), community (COMMUN), and national level (NATLHIST) information. The code for data cleaning and analysis is available from the corresponding author. The individual-level survey (PERS) records age, sex, highest level of education, and (for those who have migrated) the year of the first U.S. migration. We expanded these data into long (person-year) format starting at age 15. About 88% of individuals have middle school or less (which is completed by age 15). For the remaining 12%, we assumed a linear progression in education from age 15 onward to the final degree reported.

The household-level (HOUSE) survey documented up to six property holdings and up to four business holdings for each household. We aggregated the number of rooms owned in all properties and created a categorical indicator where 0 indicated no properties, and values of 1 to 3 corresponded to the tertiles of positive values. We coded business ownership as binary. The survey included information on purchase dates, which allowed us to project household assets backward in time. This approach misses any assets owned in the past but sold or lost before the survey date.

We use time-varying household and community information to create a pseudopanel dataset including individuals from age 15 onward. We code our main outcome (undocumented U.S. migration) using information on the timing of the first trip and documentation status of the individual upon entry. We consider an alternative definition where individuals who enter with a tourist visa but report working for a wage (thus countering the provisions of the visa) are counted as undocumented.

We remove individuals from our sample after their first U.S. trip in the main analysis and consider additional trips in robustness checks. We exclude documented migrants from our sample entirely; the results are robust to their inclusion. Finally, because we are interested in the agricultural mechanism underlying the weather–migration link, we restrict our sample to rural communities where at least 50% of the labor force works in agriculture. Our data contain 425,062 person-years observed over 27 y. These observations are nested within 48,313 individuals, 12,078 households, 84 communities, and 21 states.

### Weather Data.

Our weather measures are constructed using daily gridded weather data (Daymet) obtained from ORNL DAAC (Oak Ridge National Laboratory Distributed Active Archive Center), one of the NASA Earth Observing System Data and Information System data centers (https://daymet.ornl.gov) (50). These data offer fine-grained information and complete spatial coverage by interpolating data obtained from ground stations over a “grid” (1 km^2^ in our case) (51). We obtain shape files for the communities in the MMP data, and then overlay the weather grids on community boundaries. We aggregate the weather information for each community by taking the average measure across the grids within the community boundary.

We focus on May-to-August period within each year to capture the sensitive months for corn growth. Planting for the spring-summer season, which accounts for 75% of corn production in Mexico, starts around May. Harvesting begins in September. The ideal growing conditions require temperatures around 20 ^°^C (68 ^°^F) and rainfall between 600 and 1,000 millimeters per year (35). Rainfall deficits during crop cycle limit nutrient uptake and make the plant susceptible to pests. Rainfall excesses constrain oxygen and nitrogen supply and retard root development. Cool temperatures delay germination, while hot temperatures above 30 ^°^C (86 ^°^F), especially recurring over several days, hurt pollination (52).

To capture weather shocks to corn, we construct two measures: 1) changes in May to August precipitation from historical community mean (1980 to 1990), and 2) changes in May to August temperatures (average of minimum and maximum daily temperature) from historical community norm. Both measures are normalized by community SDs in 1980 to 1990. Rather than using a predetermined threshold for ideal weather conditions for corn, we choose to compare each community to its own past self. This approach allows us to account for regional variations in growing conditions as well as farmers’ long-run adaptations to those conditions.

## Supplementary Material

Appendix 01 (PDF)

## Data Availability

All study data are available: Researchers can replicate the analysis by downloading the public MMP data and obtaining the supplemental data, aggregate weather indicators, and data cleaning and analysis code from the corresponding author. The code for generating the aggregate weather data is also available from the corresponding author, but it requires restricted-access community identifiers. These identifiers can be obtained by reaching out to the MMP team (David Lindstrom, david_lindstrom@brown.edu). Previously published data were used for this work (mmp.opr.princeton.edu).

## Footnotes

^*^There are no administrative datasets on immigrant outflows from the United States (29). Researchers use census and survey data from Mexico and the United States to estimate return migration at the aggregate level (19).
