# Supplementary material for: Weather deviations linked to undocumented migration and return between Mexico and the United States
Source: Proc Natl Acad Sci U S A. 2024 Nov 4;121(46):e2400524121. doi: 10.1073/pnas.2400524121 (PMC11572974; doi:10.1073/pnas.2400524121)
Supplement: Supplementary file 1 — Appendix 01 (PDF) [file pnas.2400524121.sapp.pdf]

PNAS

Supporting Information for

Weather deviations linked to undocumented migration and return between Mexico and the United States

Julia Li Zhu, Nancy Chau, Amanda D. Rodewald, Filiz Garip

Filiz Garip.
E-mail: fgarip@princeton.edu

This PDF file includes:

Supporting text
Figs. S1 to S4
Tables S1 to S9
SI References

Supporting Information Text

Weather and Migration. Research on weather-related migration covers both rapid-onset events (e.g., hurricanes) and gradual processes (e.g., above average temperatures). It asks three key questions: who migrates, where, and for how long. Empirical results vary by context and across sub-populations in a setting, owing to complexity of relationships as well as differences in data and methods.

Migration is not a universal response to weather stressors; it becomes an adaptation strategy in the absence of alternative means of protecting lives and livelihoods. In Mexico, for example, rainfall deficits have little impact on mobility in urban areas (1) while triggering out-migration in rural communities dependent on rain-fed agriculture (2, 3) especially in regions without irrigation (4) given lack of insurance against crop failure (5). In the United States, tornado-stricken counties experience out-migration historically while flooded regions receive in-migration likely due to investments to protect against future flooding (6). Similarly, in rural Pakistan, heat stress is more strongly associated with out-migration relative to flooding, given smaller relief efforts to address the former (7).

Those most vulnerable to weather impacts tend to be the poor, who generally have less mobility than affluent individuals. Poor rural municipalities in Mexico, for example, display lower levels of U.S. migration in years of rainfall deficit relative to better-off areas (8). Not every move is as costly as crossing the Mexico-U.S. border, however. Poor households experiencing droughts in Ethiopia (9) or Mali (10) do send migrants, but typically across low-cost short distances. In Mexico-U.S. case, established migrant networks can reduce costs of migrating and facilitate cross-border moves for poor households (11).

Migrant networks offer access to offer information, help, or accommodation, and make migration a less selective endeavour (12). Communities with established histories of U.S. migration, for instance, show a stronger migration response to droughts in Mexico (13). But social ties to migrants can also suppress weather-related mobility (14) if, for example, prior migrants send remittances to facilitate adaptation in place.

Migration is not just a function of household and community vulnerability or resources; it can be highly selective of individual characteristics such as gender, education, and occupation. In rural Ecuadorian Andes, for example, agricultural shocks are related to increased mobility for men, but not for women. By contrast, abundant rainfall is linked to reduced migration for men (presumably due to higher need for their agricultural labor), but not for women (15). In general, women seem more constrained in weather-related mobility relative to men due to restrictive social norms, limited employment options, or lower access to migrant networks (16) in most, but not all (9), settings.

Like gender, education and occupation shape economic opportunities in both origin and destination places. Less educated individuals in Mali and Senegal, for example, are more likely to be involved in weather-dependent farming and to undertake migration to support family livelihoods (17). The impact of weather shocks can reach beyond the agricultural sector. In rural Mexico, extreme heat creates knock-on effects by reducing overall demand for goods and pushing non-farm workers to resort to migration as well (18).

Liquidity constraints, social ties, and individual characteristics can influence not just who migrates in response to weather shifts, but also where. Lower costs of mobility can make internal migration a more likely response to weather shocks relative to international movement (19) as observed in out-of-village moves following extreme heat in Pakistan (7). Individuals might choose to travel shorter distances within the same country to further reduce costs of moving. In Indonesia, for example, lower-than-average temperatures and delayed monsoon onset both increase moves within a province, but not moves to other provinces (19).

Distance to a destination is not always a barrier to migration. As in the Mexico-U.S. case, established migrant networks can reduce costs of international movement and make it a likely response under weather-related threats to local livelihoods (8, 13, 18). Distance traveled can vary by gender. In Ethiopia, droughts increase the likelihood of longer distance (out-of-district) moves for men, but not for women (9).

Similar factors (community or household vulnerability and resources, social ties, and individual attributes) likely shape duration of weather-related mobility, but empirical evidence remains relatively sparse. Research finds that higher levels of exposure to weather shocks create a longer duration migration response. As an example, severely flooded regions of Louisiana, Mississippi, and Alabama received lower rates of return migration in the year following Hurricane Katrina relative to less affected areas (20). Sub-populations also varied in their return rates due to their differential exposure to flood-related damage. After Katrina, African Americans were slower to return home relative to white residents due to greater damage to their housing units on average (21).

Findings from gradual weather shifts paint a different picture, however. Areas experiencing temperature variations in Nepal, for example, display increased short-term mobility (where migrants return within the year) and reduced long-term mobility, likely due to liquidity constraints to financing the latter (22).

Weather and Migration in Mexico. Between 1960 and 2010, number of Mexican-born individuals in the United States increased by 12 million (23); unauthorized migrants comprised about half of this population (24). Weather events likely contributed to this mobility (2). Between 1980 and 2005, weather-related hazards accounted for an estimated 80% of economic loss from disasters (including geological events, like earthquakes) in Mexico; by 2000, only 10% of cultivated land was insured against such damage (25). Researchers have linked historical droughts to crop losses in the 1980s and 1990s (25, 26) and to U.S. migration from 1970s through 2000s (27). There is now growing interest in the intersection of weather extremes and migration from Mexico.

Earlier work links Mexico-U.S. migration to labor recruitment programs after World War II (28), persistent demand for

migrant labor in the United States, economic volatility in Mexico (23), as well as wage differentials, trade relations (29), and growing migrant networks between the two countries (11).

Empirical results on weather-related migration between Mexico and the United States remain mixed. First, researchers use different metrics for weather and report conflicting findings. Nawrotzki et al. (5), for example, find a positive association between rainfall deficits (relative to prior 5-year period) and U.S. migration with Mexican Census data. Riosmena et al. (8) observe a negative association between rainfall deficits (measured in terms of deviation from the long-term mean) and U.S. migration with the same data.

Studies also report conflicting patterns on how weather shocks interact with other factors. In Riosmena et al.'s (8) study on the Mexican Census, for example, the effect of rainfall deficits on U.S. migration is more pronounced in better-off municipalities. The effect of excess temperatures, by contrast, is concentrated in poor municipalities. Similarly, in Hunter et al.'s (13) analysis of the MMP data, migration prevalence in a community is positively associated with migration response to weather events. In Nawrotzki et al.'s (14) study of the same data, migration prevalence is negative linked to migration under weather shocks.

Part of the variation in findings is contextual: Weather effects can work differently across settings. But part of the variation is due to different measurement and modeling strategies. In their meta-analysis of 51 studies, Beine and Jeusette (30) identify the best practices on the latter front. Researchers obtain stronger results when their data are at higher spatial and temporal resolution (e.g., annual rather than 5-year periods) and are longitudinal.

In this study, we follow these practices. The combination of the MMP survey with the gridded weather data allow us to track migration annually and link it to community-specific weather shocks. According to Beine and Jeusette (30), researchers also report more consistent results when they capture the mechanisms underlying the weather-migration link. Here, we focus on rural communities in Mexico, where weather shocks likely impact agricultural yields.

Many studies on Mexico-U.S. migration similarly investigate rural communities, which include mostly small farmers (those with 5 hectares of land or less) with little access to agricultural technologies (improved seeds, fertilizers) or financial tools (credit, insurance) for managing weather-related risk (31). Results show that rural areas dependent on rain-fed agriculture experience increased internal and international migration after rainfall deficits (4) and decreased mobility following a rainfall surplus relative to historical patterns (13). Similarly, regions with excessive temperatures display higher U.S.-bound migration relative to those with normal temperatures (1, 18). These patterns do not hold in urban settings, which are less vulnerable to weather fluctuations (1, 2) but can still experience out-migration as a result of sudden-onset disasters (25).

The choice between internal and international mobility hinges on the possibility of mitigating the higher costs of the latter. Harmful weather events are more likely to generate U.S. migration if they occur early in the agricultural season, thus giving individuals time to plan, while those happening mid-season are likely to induce internal migration in Mexico (18). Presence of migrant networks tips the balances in favor of international mobility in Mexico (8, 13).

Weather shocks in Mexico can determine not just whether individuals will cross the U.S. border, but also how. Excessive heat (measured via warm spell duration), for example, increases the odds of undocumented migration, but it is not related to documented crossing (32). Similarly, above-average rainfall decreases undocumented mobility, but it is not associated with documented trips.

Linking Weather to Migration. Weather-related shocks can induce migration through several mechanisms such as reducing agricultural livelihoods (2), destroying livable spaces (25) and creating knock-on effects on non-agricultural sectors (18). Studies measure these mechanisms directly or infer them by restricting the sample or time frame.

Feng et al. (2) rely on state-level data from Mexico to show weather-related annual declines in agricultural yields and resulting increase in U.S.-bound migration. Hunter et al. (13) use more granular household-level data from the same setting. By restricting the sample to rural communities, the authors suggest (not directly test) an agricultural link between weather extremes in a year and subsequent migration behavior. Jessoe et al. (18) take this approach a step further by measuring weather during the growing season for maize (the main crop in rural Mexico) and connecting it individual migration decisions. We follow both approaches here. We restrict our analysis to corn-growing rural communities, and we measure weather conditions during corn season (May to August).

Studies employ different weather measures. Some consider crop-specific vulnerabilities. Jessoe et al. (18) count days with optimal temperatures for maize. Other studies rely on more generic measures, such as average rainfall or temperature (33). Molina et al. (34) show that both kinds of measures can be equally predictive of migration choices from rural Mexico to the United States. Studies also use level-based or anomaly-based measures. Level-based measures, such as average temperature, presume weather works similarly across settings. But an average temperature of 25°C can have a different impact in Tlaxcala, one of the cooler states in Mexico, than it does in Tabasco, the warmest state, where such temperature is common. Anomaly-based measures, such as temperature deviation from the state norm, account for such variation. In this study, we take the latter approach.

An important decision is on how to calibrate weather each year with respect to its historical patterns. Studies can pick long-term (e.g., 1960-1990 in (8) or short-term windows (e.g., 1980-84 in (5)) for computing weather deviations. Here, we use a short-term window (1980-1990) to account for more recent adaptation to weather conditions.

Studies model weather effects in different ways. The most common approach is to use a single-year lag where a weather event in year t-1 is considered a potential cause of migration decisions in year t (13). We follow this approach here.

Implications of the Fixed Effects Specification. We model undocumented migration with a state and year fixed effects specification to avoid omitted variable bias from unobserved state- or time-specific factors. Because fixed effects remove any variation over

Table S1. Descriptive statistics

Variables	Mean	Standard deviation	Standard deviation (residual after FE)
Precipitation deviation	-0.04	1.05	0.95
Temperature deviation	0.27	1.27	1.06
Age	33.21	14.45	14.31
Male	0.44		
Education (yrs)	7.17	4.15	4.05
Owns land	0.31		
Owns business	0.20		
Properties: None	0.29		
Properties: Low	0.33		
Properties: Medium	0.26		
Properties: High	0.12		
Any US migrants in household	0.29		
Share of men in agriculture in community	0.64	0.10	0.12
Share ever US migrant in community	0.14	0.12	0.10
Log of community distance to US border	13.59	0.46	0.16
N (person-years)	425,062		
N (persons)	48,313		
N (households)	12,078		
N (communities)	84		
N (states)	21		
N (years)	27		

Standard errors are not reported for binary indicators. The last column presents standard errors from residual data after the indicator is regressed on state and year fixed effects. Precipitation (temperature) deviation equals May-August rainfall (average temperature) in a community in a year minus the mean value in community in 1980-1990, divided by the standard deviation in that period.

time and between states, the resulting coefficients represent the average effect of variables within a state after controlling for temporal trends. This specification reduces some of the variation in our data. Table S1 lists the standard deviations for continuous variables in the raw data (column 2) and residualized data (column 3) obtained by regressing each variable on state and year dummies and differencing actual and fitted values. The standard deviation for the continuous precipitation deviation indicator, for example, drops from 1.05 to 0.95, a reduction of 10%. The standard deviation for the temperature indicator declines from 1.27 to 1.06, by 17%. Despite these reductions, the fixed effects specification leaves intact considerable variation in our data.

Figures S1 and S2 respectively show the distribution of precipitation deviation and temperature deviation between May and August (the primary corn season). The dashed black (solid red) line corresponds to the distribution in the raw (residualized) data. Residualized data are obtained by regressing continuous indicators of weather (precipitation and temperature) deviations on state and year dummies and computing the difference between actual and fitted values. Weather deviations are based on the distance between the value for a community each year and the average value for that community in 1980-1990, divided by the standard deviation in the same period.

In both figures, the distribution of the weather indicator is slightly wider in the original data (dashed black line) relative to its distribution in the residualized data (solid red line). In both cases, the distribution of the indicator peaks at zero, meaning that most communities experience no weather deviations from their 1980-1990 normal. Because the fixed effects specification removes any state-specific changes (and thus, any weather deviations that uniformly affect all communities in a state), the peak of the distribution is higher in the residualized data. But there are still many communities experiencing extreme weather conditions as evidenced by the long tails of the distribution in the residualized data.

Table S2 shows the number of community-years that fall into weather deviation categories in the original and residualized data. Very wet (very dry) indicates precipitation in a community-year was over two standard deviations higher (lower) than its average value in the baseline period; wet (dry) shows the measure was one-to-two standard deviations higher (lower) from the average; normal means rainfall remained within one standard deviation of its baseline average. Temperature categories are computed in the same way. About 71% of community-years fall into 'normal' precipitation category in the residualized data compared to 66% of community-years in the original data. Similarly, 69% of community-years fall into 'normal' temperature category in the residualized data compared to 60% of community-years in the original data. As expected, fewer community-years experience extreme weather deviations when state-specific and temporal changes are regressed out. But there is still a good number of community-years in the residualized data that are categorized as dry or very dry (N = 139, 14%), or hot or very hot (N= 148, 15%).

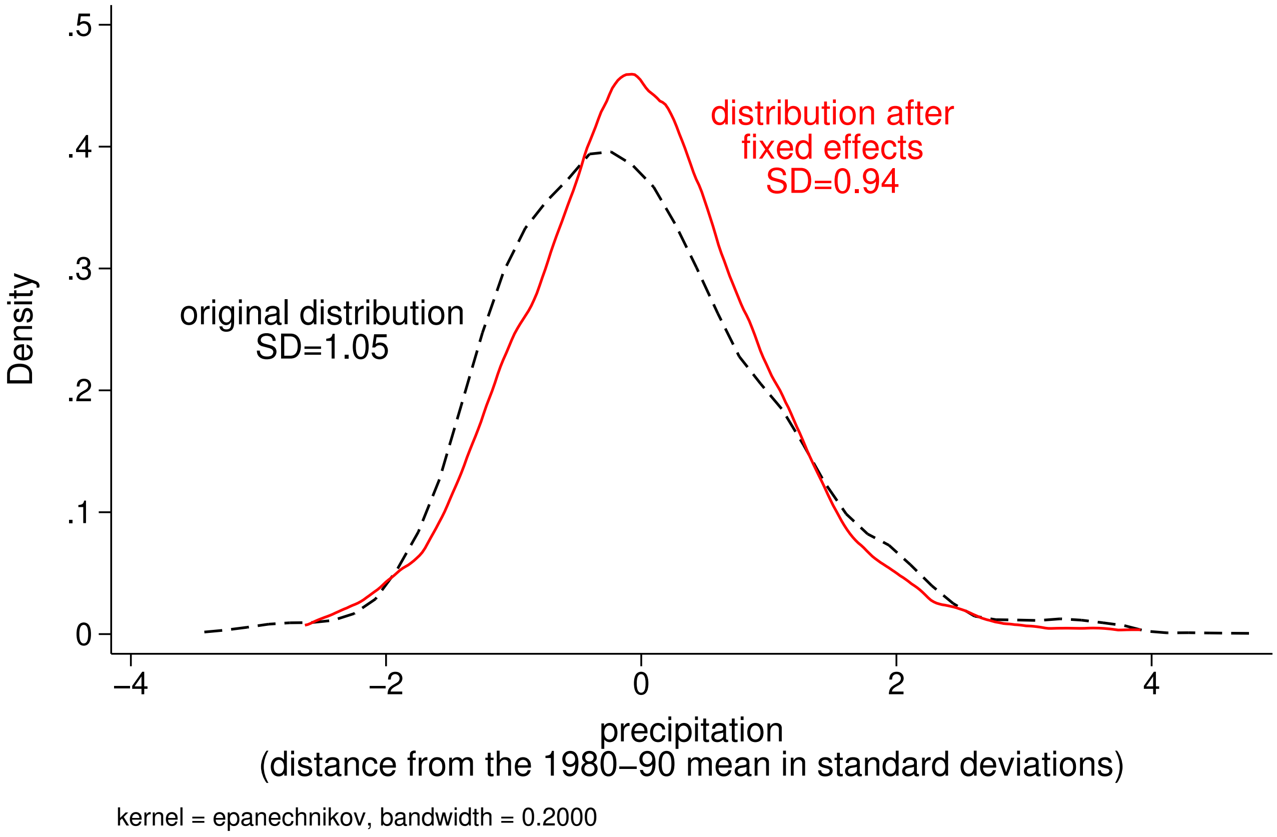

Fig. S1. The Kernel distribution of precipitation in raw and residualized data. Precipitation (total rainfall to a community in May-August) is measured with distance from the 1980-1990 mean divided by the standard deviation in the same period. The black dashed line indicates the raw data, the red solid line shows data obtained after residualizing with respect to state and year dummies.

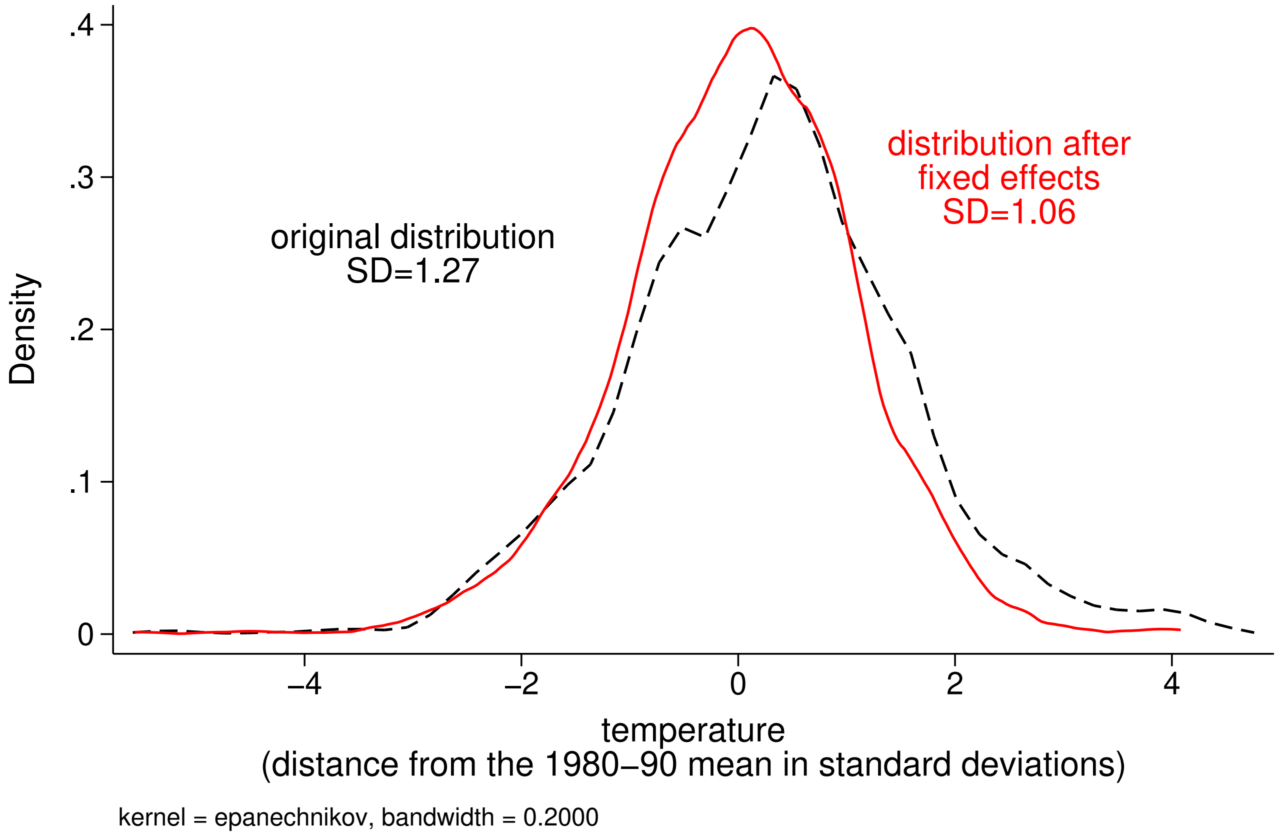

Fig. S2. The Kernel distribution of temperature in raw and residualized data. Temperature (average daily temperature in community in May-August) is measured with distance from the 1980-1990 mean divided by the standard deviation in the same period. The black dashed line indicates the raw data, the red solid line shows data obtained after residualizing with respect to state and year dummies.

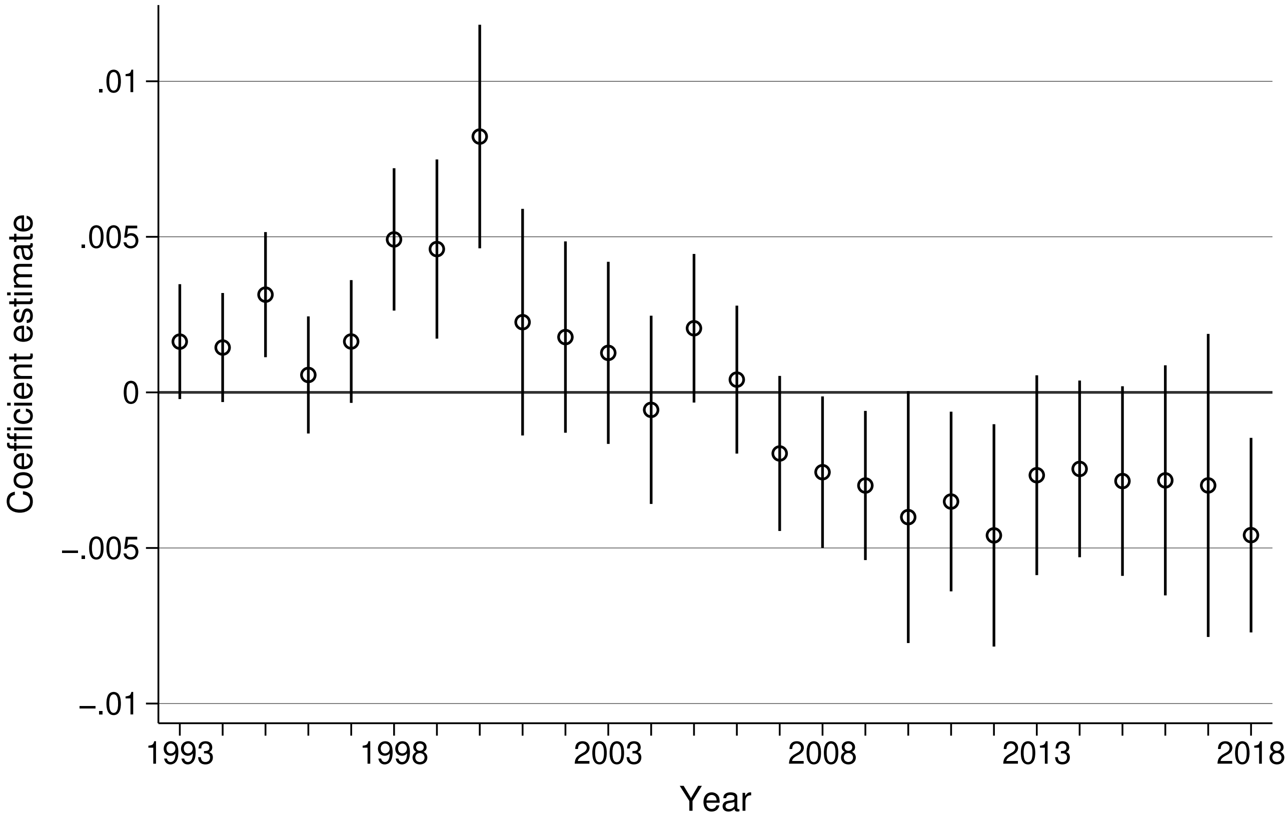

Fig. S3. OLS coefficient estimates for year dummy variables from models of first undocumented migration. Models control for weather (categories of deviations in precipitation and temperature), individual (age, sex, education), household (land, business, and property ownership, prior US migrants) and community characteristics (share in agriculture, share of ever migrants, distance to US border) as well as state dummies.

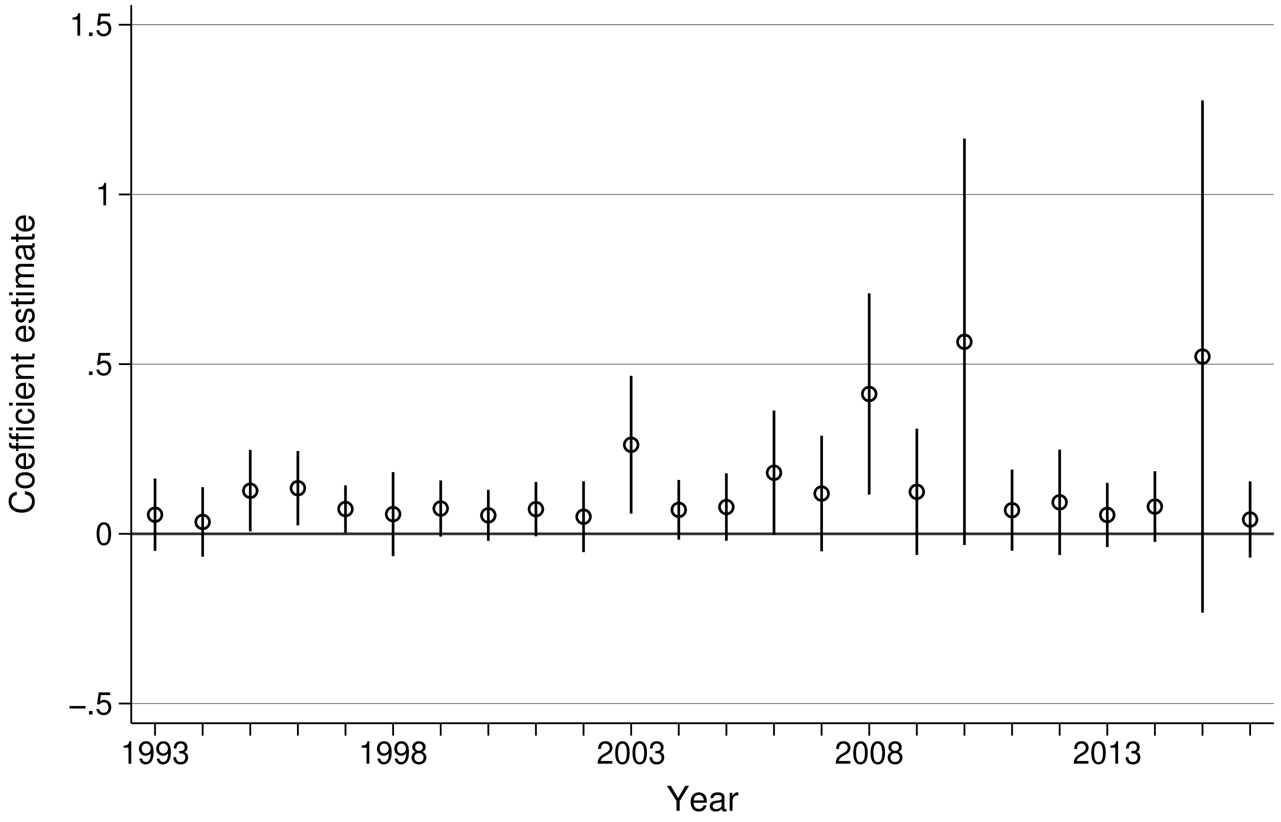

Fig. S4. OLS coefficient estimates for year dummy variables from models of return within second year in the US. Models control for weather (categories of deviations in precipitation and temperature), individual (age, sex, education, US occupation), household (land, business, and property ownership, prior US migrants) and community characteristics (share in agriculture, share of ever migrants, distance to US border) as well as Mexican state and US state dummies.

Table S2. Distribution of weather conditions across community-years

Weather condition	Community-years experiencing the weather condition			
	Raw data		Residualized data	
Very dry	15	2%	17	2%
Dry	161	17%	122	13%
Normal	641	66%	689	71%
Wet	112	11%	116	12%
Very wet	36	4%	21	2%
Very cool	42	4%	43	4%
Cool	107	11%	109	11%
Normal	579	60%	665	69%
Hot	174	18%	126	13%
Very hot	63	7%	22	2%

A community is very wet (very dry) if rainfall is over two standard deviations higher (lower) than its baseline (1980-1990) mean, wet (dry) if rainfall is one-to-two standard deviations higher (lower), and normal otherwise. Residualized data are obtained by regressing weather deviations on state and year dummies and categorizing with respect to the residuals.

Table S3. Full coefficient estimates from linear probability models of first undocumented migration and return within second year in the US in agricultural communities in the MMP data

Variables	(1) Undocumented migration	(2) Return in year 2
Age	-0.0004**	0.0025**
	(0.0000)	(0.0008)
Male	0.0121**	0.0155
	(0.0011)	(0.0145)
Education (yrs)	-0.0003**	-0.0012
	(0.0001)	(0.0019)
Owns land	-0.0004	-0.0073
	(0.0005)	(0.0135)
Owns business	-0.0010*	-0.0025
	(0.0005)	(0.0173)
Properties: Low	-0.0018**	0.0025
	(0.0005)	(0.0170)
Properties: Medium	-0.0016**	0.0016
	(0.0005)	(0.0151)
Properties: High	-0.0007	0.0020
	(0.0007)	(0.0227)
Any US migrant in household	0.0081**	0.0013
	(0.0007)	(0.0114)
Share in agriculture in community	0.0033	-0.0836
	(0.0062)	(0.0895)
Share ever US migrant in community	0.0283**	-0.0457
	(0.0055)	(0.0919)
Log of community distance to US border	0.0008	0.2749**
	(0.0048)	(0.0816)
Worked in agriculture in US		-0.0104
		(0.0187)
Migrated to California		0.0073
		(0.0150)
Migrated to Illinois		0.0302
		(0.0419)
Migrated to Texas		-0.0328
		(0.0183)
Constant	0.0064	-3.4096**
	(0.0684)	(1.0027)
N (person-years)	425,062	2,765
R^2	0.0124	0.1461

** p$<$0.01, * p$<$0.05

Robust standard errors in parentheses. Individuals with no properties are the reference group.

Table S4. Coefficient estimates for weather indicators from linear probability models of return migration within first year in the U.S in the MMP data

Weather last year	(1) Weather in season (May-Aug)	(2) Weather outside season ($<$May or $>$Aug)	(3) Temperature measured in GDD	(4) Sample includes working tourists	(5) Sample includes documented migrants
Very dry	-0.0969*	0.1634	-0.0956*	-0.1022*	-0.1056*
	(0.0408)	(0.1997)	(0.0403)	(0.0393)	(0.0452)
Dry	0.0438	-0.0095	0.0442	0.0311	0.0334
	(0.0305)	(0.0295)	(0.0305)	(0.0312)	(0.0298)
Wet	-0.0804*	-0.0264	-0.0804*	-0.0865**	-0.1021**
	(0.0310)	(0.0348)	(0.0311)	(0.0311)	(0.0312)
Very wet	-0.0879**	-0.0594	-0.0879**	-0.1086**	-0.1120**
	(0.0306)	(0.0349)	(0.0306)	(0.0323)	(0.0294)
Very cool	0.0254	0.0035	0.0247	0.0234	0.0283
	(0.0341)	(0.0460)	(0.0337)	(0.0338)	(0.0326)
Cool	-0.0110	-0.0521	-0.0112	-0.0037	-0.0067
	(0.0266)	(0.0324)	(0.0266)	(0.0250)	(0.0240)
Hot	0.0045	-0.0444	0.0033	-0.0007	-0.0027
	(0.0268)	(0.0240)	(0.0268)	(0.0267)	(0.0246)
Very hot	0.0456	0.0125	0.0361	0.0378	0.0360
	(0.0507)	(0.0290)	(0.0507)	(0.0516)	(0.0524)
N (persons)	3,427	3,427	3,427	3,595	3,897
R^2	0.1837	0.1816	0.1834	0.1868	0.2110

** $p<0.01$, * $p<0.05$. GDD = Growing Degree Days.

Robust standard errors in parentheses. Normal weather is the reference. A community is very wet (very dry) if rainfall is over two standard deviations higher (lower) than its baseline (1980-1990) mean, wet (dry) if rainfall is one-to-two standard deviations higher (lower), and normal otherwise. Models control for individual (age, sex, education, U.S. occupation), household (land, business, and property ownership, prior US migrants) and community characteristics (share in agriculture, share of ever migrants, distance to US border) as well as Mexican state, U.S. state and year dummies.

Table S5. Coefficient estimates for weather indicators from linear probability models of first undocumented migration estimated on communities with varying agricultural involvement in the MMP data

Weather last year	All communities	Share working in agriculture > 0.3	> 0.4	> 0.5
Very dry	0.0019**	0.0020*	0.0022*	0.0030*
	(0.0007)	(0.0010)	(0.0010)	(0.0013)
Dry	0.0002	0.0002	0.0002	0.0000
	(0.0003)	(0.0004)	(0.0005)	(0.0006)
Wet	-0.0001	0.0000	0.0001	0.0001
	(0.0004)	(0.0006)	(0.0006)	(0.0008)
Very wet	0.0007	0.0009	0.0014	0.0018
	(0.0007)	(0.0008)	(0.0008)	(0.0010)
Very cool	-0.0000	-0.0002	-0.0006	-0.0007
	(0.0007)	(0.0008)	(0.0009)	(0.0012)
Cool	0.0004	0.0004	0.0002	0.0005
	(0.0004)	(0.0006)	(0.0007)	(0.0008)
Hot	-0.0003	-0.0006	-0.0004	0.0001
	(0.0004)	(0.0005)	(0.0005)	(0.0006)
Very hot	0.0004	0.0000	0.0002	0.0004
	(0.0006)	(0.0009)	(0.0010)	(0.0013)
N (person-years)	976,525	712,409	586,970	425,062
R^2	0.0108	0.0116	0.0121	0.0124

** $p<0.01$, * $p<0.05$

Robust standard errors in parentheses. Normal weather is the reference.
A community is very wet (very dry) if rainfall is over two standard deviations higher (lower) than its baseline (1980-1990) mean, wet (dry) if rainfall is one-to-two standard deviations higher (lower), and normal otherwise. Models control for individual (age, sex, education), household (land, business, and property ownership, prior US migrants) and community characteristics (share in agriculture, share of ever migrants, distance to US border) as well as state and year dummies.

Table S6. Coefficient estimates for weather indicators from linear probability models of return migration within second year in the U.S. estimated on communities with varying agricultural involvement in the MMP data

Weather last year	All communities	Share working in agriculture > 0.3	> 0.4	> 0.5
Very dry	-0.0927**	-0.0870**	-0.1106**	-0.1197**
	(0.0279)	(0.0258)	(0.0311)	(0.0307)
Dry	-0.0128	-0.0155	-0.0301	0.0115
	(0.0253)	(0.0266)	(0.0308)	(0.0380)
Wet	-0.0856**	-0.0697*	-0.1010**	-0.0896**
	(0.0269)	(0.0311)	(0.0267)	(0.0289)
Very wet	-0.1251**	-0.1069**	-0.1243**	-0.0805*
	(0.0284)	(0.0294)	(0.0328)	(0.0335)
Very cool	0.1302	0.1629	0.1095	0.0366
	(0.0875)	(0.0984)	(0.0952)	(0.0722)
Cool	0.0305	0.0296	0.0289	-0.0098
	(0.0270)	(0.0240)	(0.0265)	(0.0217)
Hot	0.0220	0.0303	0.0264	0.0114
	(0.0266)	(0.0279)	(0.0306)	(0.0302)
Very hot	0.0730	0.1011	0.1069	0.0949
	(0.0528)	(0.0568)	(0.0607)	(0.0646)
N (persons)	4,724	3,988	3,513	2,765
R^2	0.1020	0.1194	0.1274	0.1461

** $p<0.01$, * $p<0.05$

Robust standard errors in parentheses. Normal weather is the reference.
A community is very wet (very dry) if rainfall is over two standard deviations higher (lower) than its baseline (1980-1990) mean, wet (dry) if rainfall is one-to-two standard deviations higher (lower), and normal otherwise. Models control for individual (age, sex, education, U.S. occupation), household (land, business, and property ownership, prior US migrants) and community characteristics (share in agriculture, share of ever migrants, distance to US border) as well as Mexican state, U.S. state and year dummies.

Table S7. Coefficient estimates for weather indicators from linear probability models of first undocumented migration estimated on municipalities with varying irrigation availability in the MMP data

Weather	Share land irrigated				
last year	> 0	> 0.1	> 0.15	> 0.2	> 0.25
Very dry	0.0050*	0.0032*	0.0029	0.0027	0.0023
	(0.0023)	(0.0014)	(0.0015)	(0.0017)	(0.0017)
Dry	-0.0004	-0.0002	-0.0005	-0.0009	-0.0012
	(0.0010)	(0.0011)	(0.0013)	(0.0012)	(0.0013)
Wet	-0.0000	-0.0003	-0.0011	-0.0014	-0.0013
	(0.0011)	(0.0013)	(0.0014)	(0.0016)	(0.0017)
Very wet	0.0019	0.0026	0.0016	0.0013	0.0015
	(0.0014)	(0.0016)	(0.0016)	(0.0016)	(0.0017)
Very cool	-0.0020	-0.0016	-0.0012	-0.0014	-0.0009
	(0.0016)	(0.0016)	(0.0018)	(0.0018)	(0.0019)
Cool	0.0018	0.0019	0.0008	0.0004	0.0008
	(0.0011)	(0.0012)	(0.0012)	(0.0013)	(0.0014)
Hot	0.0002	-0.0002	0.0002	0.0001	0.0002
	(0.0008)	(0.0009)	(0.0010)	(0.0009)	(0.0010)
Very hot	0.0012	0.0013	0.0023	0.0023	0.0023
	(0.0016)	(0.0018)	(0.0018)	(0.0018)	(0.0018)
N (person-years)	210,413	183,275	163,891	158,129	151,662
R^2	0.0121	0.0129	0.0131	0.0132	0.0133

** p<0.01, * p<0.05

Robust standard errors in parentheses. Normal weather is the reference. A community is very wet (very dry) if rainfall is over two standard deviations higher (lower) than its baseline (1980-1990) mean, wet (dry) if rainfall is one-to-two standard deviations higher (lower), and normal otherwise. Models control for individual (age, sex, education), household (land, business, and property ownership, prior US migrants) and community characteristics (share in agriculture, share of ever migrants, distance to US border) as well as state and year dummies.

Table S8. Coefficient estimates for weather indicators from linear probability models of return migration within second year in the U.S. estimated on municipalities with varying irrigation availability in the MMP data

Weather	Share land irrigated				
last year	>0	>0.1	>0.15	>0.2	>0.25
Very dry	-0.1336*	-0.1407*	-0.1419	-0.1115	-0.1206
	(0.0587)	(0.0661)	(0.0732)	(0.0702)	(0.0709)
Dry	0.0230	0.0190	0.0237	0.0440	0.0507
	(0.0466)	(0.0469)	(0.0521)	(0.0521)	(0.0524)
Wet	-0.1049*	-0.0925*	-0.1147*	-0.0730	-0.0828
	(0.0427)	(0.0454)	(0.0531)	(0.0414)	(0.0440)
Very wet	-0.1289*	-0.1300*	-0.1542*	-0.1364	-0.1440*
	(0.0587)	(0.0629)	(0.0656)	(0.0670)	(0.0669)
Very cool	0.1085	0.1228	0.1184	0.1380	0.1485
	(0.1230)	(0.1340)	(0.1469)	(0.1434)	(0.1509)
Cool	-0.0280	-0.0241	-0.0369	-0.0199	-0.0068
	(0.0362)	(0.0363)	(0.0425)	(0.0421)	(0.0364)
Hot	0.0398	0.0593	0.0572	0.0687	0.0382
	(0.0465)	(0.0515)	(0.0540)	(0.0537)	(0.0491)
Very hot	0.0713	0.0869	0.1001	0.0925	0.0810
	(0.1095)	(0.1204)	(0.1232)	(0.1206)	(0.1174)
N (person-years)	1,396	1,241	1,141	1,106	1,065
R^2	0.1660	0.1407	0.1481	0.1511	0.1586

** p$<$0.01, * p$<$0.05

Robust standard errors in parentheses. Normal weather is the reference. A community is very wet (very dry) if rainfall is over two standard deviations higher (lower) than its baseline (1980-1990) mean, wet (dry) if rainfall is one-to-two standard deviations higher (lower), and normal otherwise. Models control for individual (age, sex, education, US occupation), household (land, business, and property ownership, prior US migrants) and community characteristics (share in agriculture, share of ever migrants, distance to US border)as well as Mexican state, US state and year dummies.

Table S9. Coefficient estimates for weather indicators from linear probability models of any undocumented migration and return within second year in the US in agricultural communities in the MMP data

Variables	(1) Undocumented migration	(2) Return in year 2
Very dry	0.0027	-0.1149*
	(0.0014)	(0.0456)
Dry	0.0003	0.0410
	(0.0008)	(0.0364)
Wet	-0.0003	-0.1372**
	(0.0009)	(0.0364)
Very wet	0.0020	-0.0789*
	(0.0012)	(0.0371)
Very cool	-0.0005	0.0693
	(0.0014)	(0.0682)
Cool	0.0009	-0.0392
	(0.0009)	(0.0227)
Hot	0.0012	0.0184
	(0.0008)	(0.0318)
Very hot	0.0023	0.0872
	(0.0016)	(0.0662)
Migrated before	0.0020	-0.3285**
	(0.0012)	(0.0236)
N (person-years)	483,361	3,654
R^2	0.0124	0.2032

** p$<$0.01, * p$<$0.05

Robust standard errors in parentheses. Normal weather is the reference.
A community is very wet (very dry) if rainfall is over two standard deviations higher (lower) than its baseline (1980-1990) mean, wet (dry) if rainfall is one-to-two standard deviations higher (lower), and normal otherwise.
Models control for individual (age, sex, education), household (land, business, and property ownership, prior US migrants) and community characteristics (share in agriculture, share of ever migrants, distance to) US border as well as Mexican state, and year dummies. Return model also includes dummies for US occupation and US states.

References

1. RJ Nawrotzki, LM Hunter, DM Runfola, F Riosmena, Climate change as a migration driver from rural and urban Mexico. *Environ. Res. Lett.* **10**, 114023 (2015) Publisher: IOP Publishing.
2. S Feng, AB Krueger, M Oppenheimer, Linkages among climate change, crop yields and Mexico–US cross-border migration. *Proc. Natl. Acad. Sci.* **107**, 14257–14262 (2010) Publisher: Proceedings of the National Academy of Sciences.
3. S Feng, M Oppenheimer, Applying statistical models to the climate–migration relationship. *Proc. Natl. Acad. Sci.* **109**, E2915–E2915 (2012) Publisher: Proceedings of the National Academy of Sciences.
4. S Leyk, D Runfola, RJ Nawrotzki, LM Hunter, F Riosmena, Internal and International Mobility as Adaptation to Climatic Variability in Contemporary Mexico: Evidence from the Integration of Census and Satellite Data. *Population, Space Place* **23**, e2047 (2017) _eprint: https://onlinelibrary.wiley.com/doi/pdf/10.1002/psp.2047.
5. RJ Nawrotzki, F Riosmena, LM Hunter, Do Rainfall Deficits Predict U.S.-Bound Migration from Rural Mexico? Evidence from the Mexican Census. *Popul. Res. Policy Rev.* **32**, 129–158 (2013).
6. LP Boustan, ME Kahn, PW Rhode, Moving to Higher Ground: Migration Response to Natural Disasters in the Early Twentieth Century. *Am. Econ. Rev.* **102**, 238–244 (2012).
7. V Mueller, C Gray, K Kosec, Heat stress increases long-term human migration in rural Pakistan. *Nat. Clim. Chang.* **4**, 182–185 (2014).
8. F Riosmena, R Nawrotzki, L Hunter, Climate Migration at the Height and End of the Great Mexican Emigration Era. *Popul. development review* **44**, 455–488 (2018).
9. C Gray, V Mueller, Drought and Population Mobility in Rural Ethiopia. *World Dev.* **40**, 134–145 (2012).
10. SE Findley, Does Drought Increase Migration? A Study of Migration from Rural Mali during the 1983–1985 Drought. *Int. Migr. Rev.* **28**, 539–553 (1994) Publisher: SAGE Publications Inc.
11. DS Massey, KE Espinosa, What's Driving Mexico-U.S. Migration? A Theoretical, Empirical, and Policy Analysis. *Am. J. Sociol.* **102**, 939–999 (1997).
12. DS Massey, L Goldring, J Durand, Continuities in Transnational Migration: An Analysis of Nineteen Mexican Communities. *Am. J. Sociol.* **99**, 1492–1533 (1994).
13. LM Hunter, S Murray, F Riosmena, Rainfall Patterns and U.S. Migration from Rural Mexico. *Int. Migr. Rev.* **47**, 874–909 (2013) _eprint: https://onlinelibrary.wiley.com/doi/pdf/10.1111/imre.12051.
14. RJ Nawrotzki, F Riosmena, LM Hunter, DM Runfola, Amplification or suppression: Social networks and the climate change—migration association in rural Mexico. *Glob. Environ. Chang.* **35**, 463–474 (2015).
15. CL Gray, Gender, Natural Capital, and Migration in the Southern Ecuadorian Andes. *Environ. Plan. A: Econ. Space* **42**, 678–696 (2010) Publisher: SAGE Publications Ltd.
16. K Schmidt, Social Inequality and International Migration Related to Climate Stressors: The Case of Mexico in *Environmental Migration and Social Inequality*, Advances in Global Change Research, eds. R McLeman, J Schade, T Faist. (Springer) No. 61, (2016).
17. V van der Land, D Hummel, Vulnerability and the Role of Education in Environmentally Induced Migration in Mali and Senegal. *Ecol. Soc.* **18** (2013) Publisher: Resilience Alliance Inc.
18. K Jessoe, DT Manning, JE Taylor, Climate Change and Labour Allocation in Rural Mexico: Evidence from Annual Fluctuations in Weather. *The Econ. J.* **128**, 230–261 (2018).
19. BC Thiede, CL Gray, Heterogeneous climate effects on human migration in Indonesia. *Popul. Environ.* **39**, 147–172 (2017).
20. JA Groen, AE Polivka, Going home after Hurricane Katrina: Determinants of return migration and changes in affected areas. *Demography* **47**, 821–844 (2010).
21. E Fussell, N Sastry, M VanLandingham, Race, socioeconomic status, and return migration to New Orleans after Hurricane Katrina. *Popul. Environ.* **31**, 20–42 (2010).
22. NE Williams, C Gray, Spatial and temporal dimensions of weather shocks and migration in Nepal. *Popul. Environ.* **41**, 286–305 (2020).
23. F Garip, *On the move: Changing mechanisms of Mexico-U.S. migration*, Princeton analytical sociology series. (Princeton: Princeton University Press), (2016).
24. JS Passel, D Cohn, A Gonzalez-Barrera, Net Migration from Mexico Falls to Zero—and Perhaps Less (2012).
25. SO Saldaña-Zorrilla, K Sandberg, Impact of climate-related disasters on human migration in Mexico: a spatial model. *Clim. Chang.* **96**, 97 (2009).
26. DM Liverman, Drought Impacts in Mexico: Climate, Agriculture, Technology, and Land Tenure in Sonora and Puebla. *Annals Assoc. Am. Geogr.* **80**, 49–72 (1990) _eprint: https://onlinelibrary.wiley.com/doi/pdf/10.1111/j.1467-8306.1990.tb00003.x.
27. GN Murray-Tortarolo, MM Salgado, Drought as a driver of Mexico-US migration. *Clim. Chang.* **164**, 48 (2021).
28. DS Massey, J Durand, NJ Malone, *Beyond smoke and mirrors : Mexican immigration in an era of economic integration.* (New York: Russell Sage Foundation), (2002).
29. GH Hanson, C McIntosh, The Great Mexican Emigration. *The Rev. Econ. Stat.* **92**, 798–810 (2010).
30. M Beine, L Jeusette, A meta-analysis of the literature on climate change and migration. *J. Demogr. Econ.* **87**, 293–344 (2021).
31. B Juarez, Favorable Growing Conditions for a Higher Corn, Wheat, and Dry Beans Forecast, Sorghum Mixed, Rice Down, (USDA Foreign Agricultural Service), Technical Report MX3024 (2013).

32. RJ Nawrotzki, F Riosmena, LM Hunter, DM Runfola, Undocumented migration in response to climate change. *Int. journal population studies* **1**, 60–74 (2015).
33. R Cai, S Feng, M Oppenheimer, M Pytlikova, Climate variability and international migration: The importance of the agricultural linkage. *J. Environ. Econ. Manag.* **79**, 135–151 (2016).
34. M Molina, N Chau, AD Rodewald, F Garip, How to model the weather-migration link: a machine-learning approach to variable selection in the Mexico-U.S. context. *J. Ethn. Migr. Stud.* **0**, 1–27 (2022) Publisher: Routledge _eprint: https://doi.org/10.1080/1369183X.2022.2100549.

Mexican Ethnosurvey
of Family, Migration, and Labor

2018 to date

Universidad de Guadalajara, Princeton University
Brown University and Colegio de Mexico

Date: ______________________

Community: ______________________
Municipio: ______________________
State: ______________________

Interviewer: ______________________
Number in Census: ______________________
Random number: ______________________

For Internal Use:

Final I.D.

Community Number: ______________________

Household Number: ______________________

Observations: ______________________

TABLE A Information about the **household members and other children** of household head who no longer live in the household

(Record in this order: household head first, then the spouse; then, all the children (from oldest to youngest), lastly all other persons who live in the household.

	Name	Gender	Relationship to the household head	Household member	Year of birth	Year of death	Place of birth (Municipio and State)	Marital Status [a]	Years of educ. [b]	Principal Economic Occupation	
										Occupation	Specification
1		M F	HEAD	YES				Code			
2		M F		Yes No Unk				Code			
3		M F		Yes No Unk				Code			
4		M F		Yes No Unk				Code			
5		M F		Yes No Unk				Code			
6		M F		Yes No Unk				Code			
7		M F		Yes No Unk				Code			
8		M F		Yes No Unk				Code			
9		M F		Yes No Unk				Code			
10		M F		Yes No Unk				Code			
11		M F		Yes No Unk				Code			
12		M F		Yes No Unk				Code			
13		M F		Yes No Unk				Code			
14		M F		Yes No Unk				Code			
15		M F		Yes No Unk				Code			
16		M F		Yes No Unk				Code			
17		M F		Yes No Unk				Code			
18		M F		Yes No Unk				Code			
19		M F		Yes No Unk				Code			
20		M F		Yes No Unk				Code			

Who informed? (number in "A"):

(a) Marital Status:
1 = Single
2 = Married
3 = Consensual Union
4 = Widowed
5 = Divorced
6 = Separated

(b) Guide for years of schooling completed:
Elementary = 6
Middle School = 9
Technical w/o Middle School = 9
Technical School = 12
Academic = 12
High School = 12
Teaching School w/o High School = 13
Teaching School = 16
Superior Teaching School = 18
University = 17
Adult School = 3

TABLE B **Marital History** of the Household Head

Union	Starting year	Type of Union [a]	Year ended	Cause of Dissolution [b]
1		Code		Code
2		Code		Code
3		Code		Code
4		Code		Code

(a) Type of Union:
1 = Religious
2 = Civil
3 = Consensual Union
4 = Religious & Civil

(b) Causa de finalización de la unión:
1 = Separated
2 = Divorce
3 = Spouse's death
4 = Got married

TABLE C Information abouth each person in Table A with migratory experience within Mexico

Number in "A"	Name	Number of total trips	Trip	Place of destiny (Municipio & State)	Year of arrival	How long?	Main Economic Activity		Salary (in pesos)	
							Occupation	Specification	Quantity	Unit[a]
			1st							
			Last							Code
			1st							
			Last							Code
			1st							
			Last							Code
			1st							
			Last							Code
			1st							
			Last							Code
			1st							
			Last							Code
			1st							
			Last							Code
			1st							
			Last							Code
			1st							
			Last							Code
			1st							
			Last							Code

(a) Wage Unit
1 = Hourly
2 = Daily
3 = Weely
4 = Biweekly
5 = Montly
6 = Yearly

Table D Information abouth each person in Table A with **migratory experience to the United States or Mexico**

(U.S. migration applies to MX migrants; MX migration only applies to U.S. born children who are living in Mexico at the time of the survey)

Number in A	Name	Number of total trips	Trip	Place of destinly (City & State)	Year of arrival	Married?	How long?	Documents [a]	Main Economic Occupation		Salary ($USA)	
									Occupation	Specification	Quantity	Unit[b]
			1st			Yes No Unk		Code				Code
			Last			Yes No Unk		Code				Code
			1st			Yes No Unk		Code				Code
			Last			Yes No Unk		Code				Code
			1st			Yes No Unk		Code				Code
			Last			Yes No Unk		Code				Code
			1st			Yes No Unk		Code				Code
			Last			Yes No Unk		Code				Code
			1st			Yes No Unk		Code				Code
			Last			Yes No Unk		Code				Code
			1st			Yes No Unk		Code				Code
			Last			Yes No Unk		Code				Code
			1st			Yes No Unk		Code				Code
			Last			Yes No Unk		Code				Code
			1st			Yes No Unk		Code				Code
			Last			Yes No Unk		Code				Code
			1st			Yes No Unk		Code				Code
			Last			Yes No Unk		Code				Code
			1st			Yes No Unk		Code				Code
			Last			Yes No Unk		Code				Code

(a) U.S. Documentation:

1 = Legal resident - Green card
2 = Hired - Bracero (1942-1964)
3 = Hired - H2A o H2B
4 = Temporal: Worker
5 = Temporal: Tourist (no work permit)
6 = Citizen
7 = Silva Letter
8 = Indocumented
9 = Refugee/Asylee
11 = Double citizenship (MX/US)
12 = DACA

(b) Wage Unit

1 = Hourly
2 = Daily
3 = Weely
4 = Biweekly
5 = Montly
6 = Yearly

TABLE D2 Information about each person that has applied for **legal residency and/or U.S. citizenship**. (Do not include U.S. born)

Number in A	Name	Green Card			Citizenship	
		Year applied	Year received	Sponsor[a]	Year applied	Year received
				Code		
				Code		
				Code		
				Code		
				Code		
				Code		
				Code		
				Code		
				Code		
				Code		

(a) Sponsor:

1 = Spouse
2 = Son / Daughter
3 = Father / Mother
4 = Hermano/a
5 = Amnesty - Rodino (1987-1989)
6 = Special Agricultural Worker - SAW (1987-1989)
7 = Employer
8 = Other: ____

CUADRO DACA Information about each person **who has applied for DACA**

No. en "A"	Nombre	DACA	
		Año de solicitud	Año en que la recibió

TABLE D3 Information abouth each person in Table A with **migratory experience to Canada**

Number in A	Name	Total # of trips	Temp	Place of destiny [c] (Farm, City, and Province)	Year of Arrival	Married?	How long?*	Documents [a]	Main Occupation		Wage ($Cnd)	
									Occupation	Specify	Qty	By[b]
			1st			Yes No UNK		Code				Code
			Last			Yes No UNK		Code				Code
			1st			Yes No UNK		Code				Code
			Last			Yes No UNK		Code				Code
			1st			Yes No UNK		Code				Code
			Last			Yes No UNK		Code				Code
			1st			Yes No UNK		Code				Code
			Last			Yes No UNK		Code				Code
			1st			Yes No UNK		Code				Code
			Last			Yes No UNK		Code				Code
			1st			Yes No UNK		Code				Code
			Last			Yes No UNK		Code				Code
			1st			Yes No UNK		Code				Code
			Last			Yes No UNK		Code				Code
			1st			Yes No UNK		Code				Code
			Last			Yes No UNK		Code				Code
			1st			Yes No UNK		Code				Code
			Last			Yes No UNK		Code				Code
			1st			Yes No UNK		Code				Code
			Last			Yes No UNK		Code				Code

(a) Documents:
5 = Tourist/Visitor
8 = Indocumented
9 = Refugee
10 = Labor Contract **(PTAT)**

(b) Wage frequency:
1 = Hourly
2 = Daily
3 = Weekly
4 = Biweekly
5 = Montly
6 = Yearly

(c) Provinces
(AL) Alberta
(CB) Columbia Británica
(MT) Manitoba
(NB) New Brunswick
(NE) Nueva Escocia
(OT) Ontario
(IPE) Isla Príncipe Eduardo
(QB) Quebec
(SK) Saskatchewan

*** Minimum time of stay may vary from weeks up to 8 months**

TABLE E Information about the history of business, companies or other activities **that require economic investment** from the head or spouse

Business Number	Type of Business		Year started	Year closed / sold	Financed w/ U.S. dollars?	Number of workers		Located in the U.S.?	How acquired?[b]
	Description	Code[a]				family members?	other workers?		
1		Code			Yes No Unk			Yes No Unk	Code
2		Code			Yes No Unk			Yes No Unk	Code
3		Code			Yes No Unk			Yes No Unk	Code
4		Code			Yes No Unk			Yes No Unk	Code

(a) Type of Business:
1 = Store
2 = Street Vendor
3 = Restaurant / bar
4 = Workshop
5 = Factory
6 = Middleman (buy/sell)
7 = Personal service
8 = Profesional / Technical services
9 = Other service
10 = Agriculture
11 = Cattle raising
12 = Other

(b) How acquired?
1 = Savings
2 = Loan from bank
3 = Loan from family
4 = Loan from friends
5 = Inheritance
6 = Other: ______________________

TABLE Fa Labor history of the **household head** since the year he/she was enrolled in school or first job

Year	Age	Schooling[a]	Place (Municipio/City & State)	Economic Activity		Duration (yy.mm)	Social security/ retirement[c]	Documents (In U.S.)[b]	Documents (in Canada)[b]
				Occupation	Specification				
							Code	Code	Code
							Code	Code	Code
							Code	Code	Code
							Code	Code	Code
							Code	Code	Code
							Code	Code	Code
							Code	Code	Code
							Code	Code	Code
							Code	Code	Code
							Code	Code	Code
							Code	Code	Code
							Code	Code	Code
							Code	Code	Code
							Code	Code	Code
							Code	Code	Code
							Code	Code	Code
							Code	Code	Code
							Code	Code	Code
							Code	Code	Code
							Code	Code	Code
							Code	Code	Code

(a) Schooling
1 = Preschool
2 = Elementary School
3 = Middle School
4 = High School / Technical
5 = University
6 = Teaching School w/o HS
N/A = None
Unk = Unknown

(b) U.S. Documents:
1 = Legal resident - Green card
2 = Hired - Bracero (1942-1964)
3 = Hired - H2A o H2B
4 = Temporal: Worker
5 = Temporal: Tourist (no work permit)
6 = Citizen
7 = Silva Letter
8 = Indocumented
9 = Refugee/Asylee
10 = Contrato Temporal **(PTAT)**

(c) Seguro Social:
1 = IMSS
2 = ISSSTE
3 = Seguro Popular
4 = Progresa / Oportunidad
5 = Retired / Pensioned
6 = U.S. Social Security / Pensioned
N/A = None
Unk = Unknown

Salary during last job in Mexico

Pesos: ___________________________________

Unit: Hourly Daily Weekly Biweekly Montly Yearly

TABLE Fb Labor history of the **spouse** since the year he/she was enrolled in school or first job

Year	Age	Schooling[a]	Place (Municipio/City & State)	Economic Activity		Duration (yy.mm)	Social security/ retirement[c]	Documents (In U.S.)[b]	Documents (in Canada)[b]
				Occupation	Specification				
							Code	Code	Code
							Code	Code	Code
							Code	Code	Code
							Code	Code	Code
							Code	Code	Code
							Code	Code	Code
							Code	Code	Code
							Code	Code	Code
							Code	Code	Code
							Code	Code	Code
							Code	Code	Code
							Code	Code	Code
							Code	Code	Code
							Code	Code	Code
							Code	Code	Code
							Code	Code	Code
							Code	Code	Code
							Code	Code	Code
							Code	Code	Code
							Code	Code	Code
							Code	Code	Code

(a) Schooling
1 = Preschool
2 = Elementary School
3 = Middle School
4 = High School / Technical
5 = University
6 = Teaching School w/o HS
N/A = None
Unk = Unknown

(b) U.S. Documents:
1 = Legal resident - Green card
2 = Hired - Bracero (1942-1964)
3 = Hired - H2A o H2B
4 = Temporal: Worker
5 = Temporal: Tourist (no work permit)
6 = Citizen
7 = Silva Letter
8 = Indocumented
9 = Refugee/Asylee
10 = Contrato Temporal **(PTAT)**

(c) Seguro Social:
1 = IMSS
2 = ISSSTE
3 = Seguro Popular
4 = Progresa / Oportunidad
5 = Retired / Pensioned
6 = U.S. Social Security / Pensioned
N/A = None
Unk = Unknown

Salary during last job in Mexico

Pesos: ________________________________

Unit: Hourly Daily Weekly Biweekly Montly Yearly

TABLE G Household head **family with migratory experience** to the U.S. (includes those born in U.S.)

Relationship w/ Head	Sex	Year of 1st trip	Legal Status	Year received**	Still alive?	¿Lives in U.S.?	
						Currently?	City and State
Mother			Red Ciud Undoc Other Unk		Yes No UNK	Yes No UNK	
Father			Red Ciud Undoc Other Unk		Yes No UNK	Yes No UNK	
Sibling 1	M F		Red Ciud Undoc Other Unk		Yes No UNK	Yes No UNK	
Sibling 2	M F		Red Ciud Undoc Other Unk		Yes No UNK	Yes No UNK	
Sibling 3	M F		Red Ciud Undoc Other Unk		Yes No UNK	Yes No UNK	
Sibling 4	M F		Red Ciud Undoc Other Unk		Yes No UNK	Yes No UNK	
Sibling 5	M F		Red Ciud Undoc Other Unk		Yes No UNK	Yes No UNK	
Sibling 6	M F		Red Ciud Undoc Other Unk		Yes No UNK	Yes No UNK	
Sibling 7	M F		Red Ciud Undoc Other Unk		Yes No UNK	Yes No UNK	
Sibling 8	M F		Red Ciud Undoc Other Unk		Yes No UNK	Yes No UNK	
Sibling 9	M F		Red Ciud Undoc Other Unk		Yes No UNK	Yes No UNK	
Sibling 10	M F		Red Ciud Undoc Other Unk		Yes No UNK	Yes No UNK	
Sibling 11	M F		Red Ciud Undoc Other Unk		Yes No UNK	Yes No UNK	
Sibling 12	M F		Red Ciud Undoc Other Unk		Yes No UNK	Yes No UNK	

** Only applies for Green Card Holders or Citizens
(For Undocumented and Other assign N/A)

Table H Migration experience among **extended family and friends of head** of household
(includes those born in U.S.)

Relationship to head	How many **currently** live in U.S.?	How many (others) **lived** in U.S. before?
Uncles		
Cousins		
Nieces/ nephews		
Siblings in law (from direct family)		
Children in law		
Parent in law		
Friends		

-- Confidential --
Addresses of family/ friends in U.S.:

TABLE I Information about the **house living in and other properties** owned by household head and spouse

Property Number	Type of property[a]	Material of construction[b]	Type of floor[c]	Number of rooms	Tenancy[d]	Year acquired	Year sold	Financed w/ U.S. dollars?	Located in U.S.?	How acquired?[e]
1	1	Code	Code		Code			Yes No Unk	Yes No Unk	Code
2	Code	Code	Code					Yes No Unk	Yes No Unk	Code
3	Code	Code	Code					Yes No Unk	Yes No Unk	Code
4	Code	Code	Code					Yes No Unk	Yes No Unk	Code
5	Code	Code	Code					Yes No Unk	Yes No Unk	Code
6	Code	Code	Code					Yes No Unk	Yes No Unk	Code

(a) Type of property:
1 = House of residence
2 = House owned
3 = Lot owned
4 = Business place
5 = Apartment building
6 = Apartment owned

(b) Material of construction:
1 = Adobe and tile roof (precarious)
2 = Brick and tile roof
3 = Brick and cement roof
4 = Wood

(c) Type of floor:
1 = Dirt
2 = Cement
3 = Finished (Tile/Carpet/etc.)

(d) Tenancy:
1 = Borrowed / guest
2 = Rent
3 = Own
4 = Owner by other relative
5 = Without papers
6 = Other: _________

(e) How acquired?
1 = Savings
2 = Loan from bank
3 = Loan from family
4 = Loan from friends
5 = Inheritance
6 = Other: ________________________

Table J1 House services

Service	¿Available?
Water	Yes No Unk
Electricity	Yes No Unk
Sewer	Yes No Unk
Stove	Yes No Unk
Refrigerator	Yes No Unk
Washing machine	Yes No Unk
TV	Yes No Unk
Cable or satellite	Yes No Unk
Stereo	Yes No Unk
Telephone	Yes No Unk
Cellular	Yes No Unk
Tablet / iPad	Yes No Unk
Computer	Yes No Unk
Internet	Yes No Unk

CUADRO J2 Vehicle holdings

Vehicle number	Type of vehicle[a]	Financed w/ U.S. dollars?	Bought in U.S.?	How aquired?[b]
1	Code	Yes No Unk	Yes No Unk	Code
2	Code	Yes No Unk	Yes No Unk	Code
3	Code	Yes No Unk	Yes No Unk	Code
4	Code	Yes No Unk	Yes No Unk	Code
5	Code	Yes No Unk	Yes No Unk	Code
6	Code	Yes No Unk	Yes No Unk	Code

(a) Type of vehicle
1 = Auto
2 = Pick-up/Van/Truck
3 = Bus
4 = Tractor
5 = Taxi
6 = Motorcicle
7 = Other: _______________

(b) **How acquired?**
1 = Savings
2 = Loan from bank
3 = Loan from family
4 = Loan from friends
5 = Inheritance
6 = Other: _____________________

CUADRO J3 Remittances (confidential)

Remittances from U.S.	Answer
Household receives remittances from U.S.?	Yes No Unk
If answer was Yes: Compared to your montly salary, what's the size of remittances from the U.S.?	Small Intermediate Substantial Unk

Remittances from Canada	Answer
Household receives remittances from Canada?	Yes No Unk
If answer was Yes: Compared to your montly salary, what's the size of remittances from Canada?	Small Intermediate Substantial Unk

Table K Information about the **undocumented border crossings** of the household head or another migrant from the household

Number of person in "A": ____

(Note: If the household head has no U.S. migration experience, this information must belong to another migrant who appears in Table A. The number in Table A identifies such person. No. 1 corresponds to household head only).

Crossing	Year	Crossing Place (City & State in Mexico)	Crossed with whom?[a]	Coyote			# Deportations	Succesfull crossing?
				Used coyote?	How much paid?	Who paid?[b]		
1			Code	Yes No Unk		Code		Yes No Unk
2			Code	Yes No Unk		Code		Yes No Unk
3			Code	Yes No Unk		Code		Yes No Unk
4			Code	Yes No Unk		Code		Yes No Unk
5			Code	Yes No Unk		Code		Yes No Unk
6			Code	Yes No Unk		Code		Yes No Unk
7			Code	Yes No Unk		Code		Yes No Unk
8			Code	Yes No Unk		Code		Yes No Unk
9			Code	Yes No Unk		Code		Yes No Unk
10			Code	Yes No Unk		Code		Yes No Unk
11			Code	Yes No Unk		Code		Yes No Unk
12			Code	Yes No Unk		Code		Yes No Unk
13			Code	Yes No Unk		Code		Yes No Unk
14			Code	Yes No Unk		Code		Yes No Unk
15			Code	Yes No Unk		Code		Yes No Unk
16			Code	Yes No Unk		Code		Yes No Unk
17			Code	Yes No Unk		Code		Yes No Unk
18			Code	Yes No Unk		Code		Yes No Unk
19			Code	Yes No Unk		Code		Yes No Unk
20			Code	Yes No Unk		Code		Yes No Unk
21			Code	Yes No Unk		Code		Yes No Unk
22			Code	Yes No Unk		Code		Yes No Unk
23			Code	Yes No Unk		Code		Yes No Unk
24			Code	Yes No Unk		Code		Yes No Unk
25			Code	Yes No Unk		Code		Yes No Unk

(a) Crossed with whom?
1 = Alone
2 = With Family
3 = With Friends
4 = With Family & Friends
5 = With unknown people

(b) Who paid?
1 = Self
2 = Family
3 = Employer
4 = Friends
5 = Other: ____________________

TABLE L Information about the **household head's migratory experience** to the U.S. or **another migrant** from the household.

Number of person in "A": ______

(Note: If the household head has no U.S. migration experience, this information must belong to another migrant who appears in Table A. The number in Table A identifies such person. No. 1 corresponds to household head only).

In your trips to the U.S.:		Answer	
Help received in your trips to the U.S.:		**First Trip**	**Last Trip**
Lodging from WHOM upon arrival?[a]	Code		
Did other RELATIVES lived with you in the same house? (not counting spouse and children)		Yes No Unk	Yes No Unk
Did other FELLOW HOME-COMMUNITY MEMBERS lived with you in the same house?		Yes No Unk	Yes No Unk
When needed money, who offered HELP?[a]	Code		
Financial Activity:			
Do you have BANK ACCOUNT in U.S.?			Yes No Unk
Do you have CREDIT CARD in U.S.?			Yes No Unk
Have you declared FEDERAL TAXES?			Yes No Unk
Have you ever received the Mexican CONSULAR ID CARD?			Yes No Unk
Social Relations:			
Have you participated in SPORTS organizations?			Yes No Unk
Have you participated in RELIGIOUS/SOCIAL organizations?			Yes No Unk
What kind of relations with CHICANOS?[b]		Code	
What kind of relations with BLACKS?[b]		Code	
What kind of relations with ASIANS?[b]		Code	
What kind of relations with GRINGOS?[b]		Code	
What kind of relations with LATINOS?[b]		Code	
English			
Do you speak and understand English?[c]		Code	
Do you use ENGLISH AT HOME?[d]		Code	
Do you use ENGLISH AT WORK?[d]		Code	
Do you use ENGLISH with FRIENDS?[d]		Code	
Do you use ENGLISH in your NEIGHBORHOOD? (shopping, street, etc.)[d]		Code	

(a) Who helped?
1 = Fellow home-community member
2 = Friend
3 = Employer
4 = Relative
5 = Bank
6 = Did not need help
7 = Other: _______________

(b) Type of relations:
0 = None or casual
1 = Workplace
2 = Friendship
3 = Very Close
4 = Other: _______________

(c) English
0 = Doesn't speak nor understands
1 = Doesn't speak but understands a little
2 = Doesn't speak but understands well
3 = Both: speaks and understands a little
4 = Both: speaks and understands well

(d) English usage
1 = None
2 = Sometimes
3 = Often
4 = Always

TABLE M Information about the **financial situation** on U.S. during **last trip**

(IMPORTANT: This information must correspond to the same migrant listed in Table L).

Questions		Answers		
This information refers to the most recent job of the head and spouse		***Head***	***Spouse***	***Migrant in A***
		Use these two columns if the household head is a migrant		Information obtained only if the household head is NOT a migrant
How do (did) you get your job?[a]	Code			
How much do (did) you get paid hourly ?	Dollars			
How many hours do (did) you work per week?	Quantity			
How many months per year do (did) you work?	Quantity			
Do (did) you get paid with check or cash?		C E Unk	C E Unk	C E Unk
Social security taxes withheld?		Sí No Unk	Sí No Unk	Sí No Unk
Federal taxes withheld?		Sí No Unk	Sí No Unk	Sí No Unk
Race of head's/ spouse's employer?[b]	Code			
Race of head's/ spouse's supervisor?[b]	Code			
This is about spending, savings, and remittances		***Household***		
Rent per month	Dollars			
Food budget per month	Dollars			
Average montly remittances	Dollars			
Purpose of remittances[c] (All that apply)	Codes			
Average montly savings	Dollars			
Savings brought to Mexico	Dollars			
Purpose of Savings[c] (All that apply)	Codes			

(a) How was job obtained?
1 = Searched by oneself
2 = Recommended by a relative
3 = Recommended by a friend
4 = Recommended by a fellow home-community meml
5 = Recommended by a coyote
6 = Contracted
7 = Paid a friend/ fellow home-community member
8 = Through an employment agency
9 = "Pick-up" on street corner

(b) Race:
1 = Anglo
2 = Black
3 = Asian
4 = Chicano
5 = Mexican
6 = Other Latino
7 = Other: ____________
8 = Company

(c) Remittances and savings:
1 = Food and maintenance
2 = Construction or repair of house
3 = Purchase of house or lot
4 = Purchase of vehicle
5 = Purchase of tools
6 = Purchase of livestock
7 = Purchase of agricultural inputs
8 = Purchase of consumer goods
9 = Start/expand business
10 = Education expenses
11 = Health expenses
12 = Debt payment
13 = Finance a special event
14= Recreation / entretainment
15 = Savings
16 = Other: _______________

TABLE N Information about the use of **public services** in U.S.

(IMPORTANT: This information must correspond to the same migrant listed in Table L).

During trips to the U.S.		Answers		
Have you had children in public schools?		Yes	No	Unk
Have you ever received unemployment compensation?		Yes	No	Unk
Have you ever received food stamps?		Yes	No	Unk
Have you ever received welfare?		Yes	No	Unk
What type?[a]	AFDC	Yes	No	Unk
	WIC	Yes	No	Unk
	SSI	Yes	No	Unk
	General	Yes	No	Unk
Have you or a family member gone to the doctor?		Yes	No	Unk
Have you or a family member been hospitalized?		Yes	No	Unk
If someone went to the doctor or hospital, who paid?[b]	(All that apply)			

(a) Types of welfare:

AFDC	Aid for Families with Dependent Children
WIC	Women Infants Children Program
SSI	Supplemental Security Income Program
General	State assistant (not federal)

(b) Who paid doctor/ hospital?

1 = Employer
2 = Private insurance
3 = Medicaid
4 = Self
5 = Relative
6 = No one
7 = Other: ___________________________

Table L2 Information about the **household head's migratory experience** to Canada or **another migrant** from the household. Number of person in "A": ____

(Note: If the household head has no U.S. migration experience, this information must belong to another migrant who appears in Table A. The number in Table A identifies such person. No. 1 corresponds to household head only).

**In your trips to Canada…**		Answer
Help on your first trip:		
When needed money, who offered HELP?[a]	Code	
Financial Activity:		
Do you hace BANK ACCOUNT in Canada?		Yes No Unk
Have you applied for TAX RETURN in Canada?		Yes No Unk
Social Relations:		
Have you participated in SPORTS organizations?		Yes No Unk
Have you participated in RELIGIOUS/SOCIAL organizations?		Yes No Unk
What kind of relations with BLACKS?[b]	Code	
What kind of relations with ASIANS?[b]	Code	
What kind of relations with CANADIANS?[b]	Code	
What kind of relations with LATINOS?[b]	Code	
Inglés:		
Do you speak and understand ENGLISH?[c]	Code	
Do you use ENGLISH AT WORK?[d]	Code	
Do you use ENGLISH with FRIENDS?[d]	Code	
Do you use ENGLISH at the FARM?[d]	Code	
French:		
Do you speak and understand FRENCH?[c]	Code	
Do you use FRENCH AT WORK?[d]	Code	
Do you use FRENCH with FRIENDS?[d]	Code	
Do you use FRENCH at the FARM?[d]	Code	

(a) Who helped?
1 = Fellow home-community member
2 = Friend
3 = Employer
4 = Relative
5 = Bank
6 = Did not need help
7 = Other: _______________

(b) Type of relations:
0 = None or casual
1 = Workplace
2 = Friendship
3 = Very Close
4 = Other: _______________

(c) English & French
0 = Doesn't speak nor understands
1 = Doesn't speak but understands a little
2 = Doesn't speak but understands well
3 = Both: speaks and understands a little
4 = Both: speaks and understands well

(d) English & French usage
1 = None
2 = Sometimes
3 = Often
4 = Always

Table M2 Information about the **financial situation** in Canada during **last trip**

(IMPORTANT: This information must correspond to the same migrant listed in Table L2).

Questions		Answers						
This information refers to the first job ever in Canada		**HEAD only**						
How did your get your job?[a]	Code							
How much did you get paid hourly?	$Cnd.							
How many months per year did you work?	Qty							
Did you get paid with check or cash?		Check Cash Unk						
Race of head's employer?[b]	Code							
Race of head's surpervisor?[b]	Code							
Type of farm where worked?		Family			Company			
Type of crops grown?[d]	Code		Specify:*					
This information refers to the last job in Canada		***HEAD only***						
Type of worker?[e]	Code							
How much did you get paid hourly?	$Cnd.							
How many hors did you work per week?	Qty							
How many months per year did you work?	Qty							
Did you get paid with check or cash?		Check Cash Unk						
Race of head's employer?[b]	Code							
Race of head's surpervisor?[b]	Code							
Type of farm where worked?		Family			Company			
Type of crops grown?[d]	Code		Specify:*					
This is about spending, savings, and remittances		***Household***						
Food budget per month	$Cnd.							
Average monthly remittances	$Cnd.							
Purpose of remittances[c] (All that apply)	Code							
Average monthly savings	$Cnd.							
Savings brought to Mexico	$Cnd.							
Purpose of savings[c] (All that apply)	Code							

(a) ¿Cómo obtuvo el empleo?
1 = Searched by oneself
2 = Recommended by a relative
3 = Recommended by a friend
4 = Recommended by a fellow worker
5 = Other

(d) Type of Crop
1 = Greenhouse
2 = Open land
* Specify type of crop(tomato, tobacco, etc.)

(b) Race:
1 = Canadian / European
2 = Black
3 = Asian
4 = Mexican
5 = Latino
6 = Menonite
7 = Other

(e) Type of Worker
1 = Called by employer
2 = STyPS selection

(c) Remittances and savings:
1 = Food and maintenance
2 = Construction or repair of house
3 = Purchase of house or lot
4 = Purchase of vehicle
5 = Purchase of tools
6 = Purchase of livestock
7 = Purchase of agricultural inputs
8 = Purchase of consumer goods
9 = Start/expand business
10 = Education expenses
11 = Health expenses
12 = Debt payment
13 = Finance a special event
14= Recreation / entretainment
15 = Savings
16 = Other: _______________

TABLE S **Employment Characteristics and Costs as a Temporal Worker** to the U.S. or Canada on the last trip

IMPORTANT: This information should correspond to the migrant who reported a trip to the U.S. or Canada with a H2A/H2B visas or PTAT respectively

Country where labored as a temporary worker [ ] Number of person in A: [ ]

During your last trip as temporal worker to the U.S. or Canada:	Answer				
To apply for job, did you pay a contractor or a middleman?	Yes No Unk				
How much did you pay the contractor/middleman?	$		Currency[a]:		
**Visa application on last trip**					
How much did the visa cost?	$		Currency[a]:		
Who paid for the visa application/paperwork?[b] Code					
Upon arrival, did the employer took away any of your documents?	1 = Passport	2 = Visa	3 = Both	4 = None	Unknown
**Traveling to the country - U.S./Canada**					
Who paid your trip to the U.S./Canada?[b] Code	If you paid, how much did you pay?				$
				Currency[a]:	
Who paid your return to Mexico?[b] Code	If you paid, how much did you pay?				$
				Currency[a]:	
**Contract**					
Did you receive a contract copy?	Yes No Unk				
Were the contract terms kept?	Yes No Unk				
If contract was broken, what aspects were violate (Mark all that apply)					
**Living conditions and transportation**					
Who paid for transportation from housing to work?	1 = Worker	2 = Employer	N/A	UNK	
Was housing provided	Yes No Unk				
If you paid for housing, how much did you pay?	$		Currency[a]:	Frequency:	
Did you share housing?	Yes No Unk				
How many persons shared the housing?					
Type of housing	1 = House	2 = Apartment	3 = Mobile home	4 = Other	UNK
Did the house have:	Kitchen	Dining Room	Living Room		
How many…	Bathrooms: ______		Bedrooms: _______		
How would you consider the housing condition?	Exellent	Very Good	Good	Regular	Poor

(a) **Currency**
1 = U.S. Dollar
2 = Canadian Dollar
3 = Mexican peso
8888 = There was no payment
9999 = Unknown

(b) **Who paid for trip?**
1 = Employer / contractor
2 = Worker
3 = Both: Employer and worker
4 = Family acquantaince
5 = Other ________________
9999 = Unknown

(c) **Contract violations**
1 = Incomplete work season
2 = Received less salary than promised
3 = Worked more hours than stated
4 = Overtime not paid
8888 = No violations; contract was kept
9999 = Unknown

(d) Payment frequency
1 = Daily
2 = Weekly
3 = Biweekly
4 = Monthly
8888 = Did not pay rent
9999 = Unknown

TABLE T Work Conditions - temporal workers in the U.S./Canada on last trip

IMPORTANT: This information should correspond to the same person listed in table S, and the same country

During your last trip as temporal worker	Answer
**Maltratos y accidentes laborales**	
Have you been mistreated?	Yes No Unknown
By whom?	Employer Supervisor Other N/A Unknown
Did you report it to the Embassy, Consulate or STyPS?	Yes No N/A Unknown
Did you suffer any accidents/illnesses due to work labor?	Yes No Unknown
Did you receive medical treatment?	Yes No N/A Unknown
Who paid for medical treatment?[a] Código	
**Work conditions**	
Were there any other local workers (whites, afroamericans, asians) at your work?	Yes No Unknown
Were there any other latinos at your work?	Yes No Unknown
Did they do the same work that you did?	Yes No Unknown
Did they receive the same payment as you did?	Yes No Unknown
If worked overtime, did you get paid overtime?	Yes No Unknown
If you got paid overtime, what was the payment rate?	Regular Rate Overtime N/A Unknown
Did you work the full season?	Yes No Unknown
Have you ever received unemployment benefits?	Yes No Unknown
Have you ever asked for help to the Mexican Consulate or Embassy in the U.S./Canada?	Yes No Unknown
Did you have any experience with labor unions?	Yes No Unknown

(a) Who paid for medical treatment?

1 = Employer/supervisor
2 = Worker (self)
3 = Family or friend
4 = Private Insurance
5 = Public Insurance
6 = Mexican consulate
7 = Other ______________
8888 = N/A - no treatment received
9999 = Unknown

TABLE O Current and past lands - agricultural lands

Land #	Number of hectares	Type of land[a]	Number of cultivated hectares	Tenency[b]	Year acquired	Year sold	Financed w/ U.S. dollars?	Located in U.S.?	How acquired?[c]
Current Land									
1		Code		Code			Yes No Unk	Yes No Unk	Code
2		Code		Code			Yes No Unk	Yes No Unk	Code
3		Code		Code			Yes No Unk	Yes No Unk	Code
4		Code		Code			Yes No Unk	Yes No Unk	Code
Past Lands									
1		Code		Code			Yes No Unk	Yes No Unk	Code
2		Code		Code			Yes No Unk	Yes No Unk	Code
3		Code		Code			Yes No Unk	Yes No Unk	Code
4		Code		Code			Yes No Unk	Yes No Unk	Code

(a) Type of land:
1 = Irrigated
2 = Wetland
3 = Dryland
4 = Grazing/pasture
5 = Orchard
6 = Other: ________________

(b) Tenency:
1 = Ejido
2 = Private
3 = Communal
4 = Leased

(c) How acquired?
1 = Savings
2 = Loan from bank
3 = Loan from family
4 = Loan from friends
5 = Inheritance
6 = Other: ____________________

TABLE P Information about the **usage of current land**

Question	Answer		
Information about the cultivated land			
Number of family members who work (head included)			
Number of non-family workers who help			
Use of agricultural machinery?	Yes	No	Unk
Use of fertilizers?	Yes	No	Unk
Use of insecticides?	Yes	No	Unk
U.S. financing to cultivate?	Yes	No	Unk

Information about animals	***How many?***	***Financed w/ U.S. dollars?***		
Cows		Yes	No	Unk
Pigs		Yes	No	Unk
Horses		Yes	No	Unk
Donkeys		Yes	No	Unk
Oxen		Yes	No	Unk
Chicken		Yes	No	Unk
Goats		Yes	No	Unk
Other: ______________________		Yes	No	Unk

TABLE Q Household head & Spouse **HEALTH**

Questions	Answers			
Information at Survey Time	**Household Head**		**Spouse**	
Height	meters		metros	
Weight	kgs		kgs	
Do you currently smoke?	Yes No Unk		Yes No Unk	
Have you ever smoked?	Yes No Unk		Yes No Unk	
Age at first smoke	years		years	
How do you consider your health?	**Household Head**		**Spouse**	
Quality of health at age 14	V G R P		V G R P	
Quality of health last year	V G R P		V G R P	
Current quality of health	V G R P		V G R P	
Have you ever had or currently have…	**Household Head**		**Spouse**	
		**Year of diagnosis**		**Year of Diagnosis**
Hypertension or high blood pressure	Yes No Unk		Yes No Unk	
Diabetes or high sugar levels	Yes No Unk		Yes No Unk	
Heart attack or heart problems	Yes No Unk		Yes No Unk	
Stroke	Yes No Unk		Yes No Unk	
Chronic lung conditions	Yes No Unk		Yes No Unk	
Emotional, nerves, or psychiatric problems	Yes No Unk		Yes No Unk	
Cancer or malignant tumor	Yes No Unk		Yes No Unk	
Only ask whenever there's been a migration trip	**Household Head**		**Spouse**	
Quality of health prior to U.S. migration	V G R P N/A		V G R P N/A	
Quality of health after U.S. migration	V G R P N/A		V G R P N/A	

V = Very Good
G = Good
R = Regular
P = Poor
N/A = Not a migrant

TABLE R Violence perception in the community by the household head and any other member of the household

In the last 12 months	… within your community, have you been affected by …						In your community, have you heard of…		
	Household head			Other family member					
Armed robbery	Yes	No	Unk	Yes	No	Unk	Yes	No	Unk
Pedestrian assault	Yes	No	Unk	Yes	No	Unk	Yes	No	Unk
Injuries, blows, or stab wounds	Yes	No	Unk	Yes	No	Unk	Yes	No	Unk
Gunshot injuries	Yes	No	Unk	Yes	No	Unk	Yes	No	Unk
Sexual offenses or rape	Yes	No	Unk	Yes	No	Unk	Yes	No	Unk
Extortion, threat or deception	Yes	No	Unk	Yes	No	Unk	Yes	No	Unk
Kidnapping or express kidnapping	Yes	No	Unk	Yes	No	Unk	Yes	No	Unk
Home robbery	Yes	No	Unk	Yes	No	Unk	Yes	No	Unk
Autoparts/ auto-tools robbery	Yes	No	Unk	Yes	No	Unk	Yes	No	Unk
Card theft card cloning, or theft at an ATM	Yes	No	Unk	Yes	No	Unk	Yes	No	Unk
Other thefts (specify up to 2):	(1)	(2)		(1)	(2)		(1)	(2)	
Other crimes (specify up to 2):	(1)	(2)		(1)	(2)		(1)	(2)	
Intimidation or threats to youth by gangs	Yes	No	Unk	Yes	No	Unk	Yes	No	Unk
Do you think theft/violence in your community is…	0=Not a problem	1=Of little concern		2=Problematic		3=Very Problematic		UNK	
